# PFKP in malignant tumors: bridging metabolic reprogramming, post-translational modifications, and the immunosuppressive microenvironment

**DOI:** 10.3389/fcell.2026.1944968

**Published:** 2026-09-18

**Authors:** Jing-Jing Zhao, Gangyuan Ma, Rui Zong, Jinlan Zhong, Yicheng Zhao, Liang Liu, Runze Li

**Affiliations:** 1 Chinese Medicine Guangdong Laboratory/State Key Laboratory of Traditional Chinese Medicine Syndrome, The Second Clinical College, Guangzhou University of Chinese Medicine (Guangdong Provincial Hospital of Chinese Medicine), Guangzhou, Guangdong, China; 2 Guangzhou Laboratory, Guangzhou, China

**Keywords:** glycolysis, metabolic reprogramming, PFKP, post-translational modification, tumor immune evasion, tumor microenvironment

## Abstract

Metabolic reprogramming is a hallmark of malignant tumors. Platelet-type phosphofructokinase (PFKP), a rate-limiting enzyme in glycolysis, is aberrantly overexpressed across diverse cancers and promotes tumor proliferation, invasion, and metastasis through both metabolic and non-metabolic pathways. The enzymatic activity, stability, and subcellular localization of PFKP are dynamically regulated by post-translational modifications—including phosphorylation, ubiquitination, acetylation, and lactylation—as well as by protein–protein interaction networks. Upon nuclear translocation, PFKP dissociates from glycolytic flux and engages in oncogenic transcriptional regulation. Moreover, PFKP upregulates PD-L1 expression, alters chemokine profiles, disrupts immune cell infiltration, and fosters an immunosuppressive microenvironment that facilitates immune evasion. Emerging evidence suggests PFKP holds diagnostic and prognostic potential, yet direct targeting evidence remains lacking, and most inhibitor studies are confined to preclinical models. This review synthesizes recent advances in PFKP research, focusing on its regulatory networks in tumor metabolism, post-translational modifications, protein interactions, and immune remodeling. We critically evaluate current controversies, unresolved questions, and translational barriers, and propose future directions—including epigenetic regulation, functional characterization of interacting partners, and combination strategies with immunotherapy—to inform precision oncology and targeted therapeutic development.

## 1 Introduction

The Warburg effect is a fundamental characteristic of tumor metabolic reprogramming. Cancer cells, even in the presence of oxygen, preferentially utilize glycolysis for energy production. This process efficiently generates ATP and supplies carbon skeletons necessary for the biosynthesis of nucleotides, amino acids, and lipids. Concurrently, the excessive production of lactic acid enhances tumor invasion and facilitates immune evasion by acidifying the tumor microenvironment and inhibiting immune cell function (Zhang et al., 2025; Zhong et al., 2022). Phosphofructokinase-1 (PFK-1) represents the rate-limiting enzyme of glycolysis and exists as three tissue-specific isoforms: platelet-type PFKP, liver-type PFKL, and muscle-type PFKM. Platelet-type phosphofructokinase (PFKP), one isoform of PFK-1, functions as the principal rate-limiting enzyme within the glycolytic pathway. It is extensively acknowledged as a fundamental molecular marker of cancer metabolic reprogramming and a pivotal mediator implicated in the tumor Warburg effect (Ishaq et al., 2024; Fukushi et al., 2022; Moreno-Sánchez et al., 2014; Kanai et al., 2019; Vora et al., 1985; Webb et al., 2017; Webb et al., 2015). This phenomenon elucidates that cancer cells sustain a vigorous glycolytic metabolism via elevated PFKP expression, even in the presence of oxygen, thereby facilitating substantial lactate production and rapid energy provision. This metabolic adaptation establishes a critical foundation for the proliferation of malignant tumors (Sukonina et al., 2019). Recent studies have not only further validated the central role of PFKP in aerobic glycolysis of tumors but also thoroughly uncovered its diverse biological functions during tumor initiation and progression. Accumulating evidence confirms that PFKP is far more than a simple metabolic catalytic enzyme; instead, it acts as a multifunctional molecule governing malignant tumor progression (Cao et al., 2026; Lang et al., 2019; Yuan et al., 2025; Ganapathy-Kanniappan, 2020).

Recent advancements in metabolic research have significantly altered the traditional perception of PFKP as merely a glycolytic rate-limiting enzyme. Through the integration of isotope tracing, metabolomics, and gene-editing technologies, contemporary studies have reestablished PFKP’s pivotal role within the tumor molecular network. Numerous seminal investigations have demonstrated that PFKP is specifically overexpressed in a variety of malignancies, including lung and breast cancers. In these contexts, PFKP not only facilitates tumor progression by functioning as the rate-limiting enzyme of glycolysis but also engages in molecular regulation as a protein kinase across diverse tumor types (Shen et al., 2020; Mo et al., 2020; Peng et al., 2024). For example, in conditions of amino acid deprivation, PFKP functions as a protein kinase to facilitate the phosphorylation of ATG4B, consequently regulating ATG4B activity and influencing autophagic flux (Li et al., 2021). Post-translational modifications (PTMs) of phosphofructokinase platelet (PFKP) and its associated protein-protein interaction networks function as central regulators of its biological activities, establishing a multi-dimensional regulatory hub that orchestrates tumor initiation, proliferation, metastasis, and therapeutic resistance. For instance, the reactivation of the epidermal growth factor receptor by Wnt3A initiates the PI3K/AKT signaling pathway, which subsequently enhances PFKP expression through phosphorylation at the S386 site. This modification promotes cell proliferation, colony formation, and the migratory capacity of cancer cells (Jeon et al., 2021). The cytoplasmic E3 ubiquitin ligase HRD1 engages with PFKP to facilitate its ubiquitination and subsequent proteolytic degradation, thereby inhibiting breast tumor proliferation and metastasis *in vivo* (Fan et al., 2021). Within tumor cells, PFKP interacts with intracellular signaling pathways through metabolite signaling, thereby regulating the adaptability of cancer cells to challenging microenvironmental conditions, such as hypoxia and nutrient deprivation. This interaction influences both the biological behaviors of cancer cells and their responses to anti-tumor immunity (Gong et al., 2024; Han et al., 2024). Significantly, seminal studies have demonstrated that PFKP serves as a pivotal mediator of the interplay between transcriptional and epigenetic regulation, functioning as a crucial molecular link between transcription and epigenetic modifications. Specifically, the direct interaction of PFKP with ERK2 activates the MAPK/ERK signaling pathway and enhances the stability of the transcription factor c-Myc. The resultant accumulation of c-Myc subsequently promotes PFKP transcription, thereby establishing a self-reinforcing positive feedback loop (Liu et al., 2024). These findings collectively indicate that PFKP serves as both a significant diagnostic biomarker and a promising therapeutic target across a wide range of human cancers.

While prior reviews have encapsulated the metabolic roles of PFKP, the majority predominantly emphasize its involvement in glycolytic regulation. There is a paucity of literature that thoroughly integrates PFKP-mediated post-translational modification networks, non-metabolic signaling pathways, and immune remodeling within the tumor microenvironment. The interactions between PFKP and the immunosuppressive microenvironment are not yet comprehensively organized, thereby constraining our understanding of its translational potential.

PFKP plays a pivotal role in tumor progression and therapeutic resistance by modulating glycolysis and various oncogenic signaling pathways across multiple cancer types. Consequently, there is a pressing need for a comprehensive synthesis and in-depth examination of the regulatory mechanisms associated with PFKP in malignancy. In this review, we elucidate the subcellular localization and diverse biological functions of PFKP, summarize the molecular mechanisms through which PFKP facilitates tumor progression, and discuss therapeutic strategies targeting PFKP. Additionally, we integrate recent findings that link PFKP to tumor immune remodeling. This work consolidates and clarifies the translational potential of PFKP, with the aim of facilitating the development of targeted interventions and improving patient prognostic outcomes.

We conducted a comprehensive literature search in PubMed and Web of Science for studies published up to July 2026. Emphasis was placed on peer-reviewed original research articles and authoritative reviews that address PFKP, tumor metabolism, post-translational modifications, and the tumor immune microenvironment. Studies deemed representative and relevant to these topics were selected and summarized.

## 2 The identification and characterization of PFKP

Phosphofructokinase-1 (PFK-1) facilitates the rate-limiting and committed step in the glycolytic pathway. Three human isoforms of PFK-1, each specific to different tissues, have been identified: PFKP (platelet-type), PFKL (liver-type), and PFKM (muscle-type). These isoforms exhibit distinct patterns of tissue distribution, enzymatic kinetics, and regulatory responsiveness (Muntz, 1953; Kahn et al., 1979; Tsai and Kemp, 1973; Vora et al., 1980). The successful cloning of full-length human PFKP cDNA in 1991 enabled comprehensive investigations into its molecular structure, regulatory mechanisms of expression, and biological functions (Simpson and Fothergill-Gilmore, 1991; Eto et al., 1994; Eto et al., 1994; NormaMorrison, 1992).

Early research typically characterized PFKP as a housekeeping enzyme responsible for maintaining cellular energy metabolic homeostasis, with functions primarily confined to the regulation of glycolysis. However, with the progression of the theory of tumor metabolic reprogramming, PFKP has been identified as a critical nexus connecting glucose metabolism with malignant phenotypes. It not only supports glycolytic flux in tumor cells but also plays a significant role in cell proliferation, invasion, and metabolic adaptation (Monika et al., 2011; Moon et al., 2011; Zancan et al., 2010).

In recent years, there has been a progressive elucidation of the non-metabolic functions of PFKP. PFKP has been shown to establish regulatory networks with a range of molecules, including microRNA-520 and PPARγ, and to participate in pivotal pathological processes such as the remodeling of the tumor microenvironment, the maintenance of cancer stem cell stemness, and the development of chemoresistance (Lincet and Icard, 2014; Calvier et al., 2017; Park et al., 2013). The high-resolution crystal structure of human PFKP tetramer resolved in 2015 enables mechanistic exploration of its quaternary assembly and allosteric regulation (Kloos et al., 2015; Webb et al., 2015). Despite sharing approximately 70% amino acid sequence homology with PFKL and PFKM, PFKP exhibits isoform-specific biochemical characteristics due to divergent residues within its subunit interfaces and allosteric pockets. The equilibrium between inactive dimers and catalytically active tetramers varies significantly among the three isoforms, with PFKP demonstrating distinct oligomer stability in comparison to PFKL and PFKM. Furthermore, the allosteric binding sites for ATP, citrate, AMP, and fructose-2,6-bisphosphate exhibit notable structural differences across the isoforms. These structural variations underpin the molecular basis for pharmacological selectivity (Lynch et al., 2024; Campos and Albrecht, 2023; Fernandes et al., 2020). Consequently, the rational design of small-molecule agents that selectively target PFKP is heavily dependent on the isoform-specific structural features of PFKP. Structural analyses further elucidate how tumor-associated mutations alter PFKP oligomerization, allosteric activation, and catalytic activity, providing a crucial theoretical foundation for the development of PFKP-targeted therapeutics. With the advancement of our comprehension of PFKP’s biological roles, research has broadened from traditional metabolic regulation to encompass epigenetic regulation. Increasing evidence suggests that PFKP influences metabolite production and signal transduction, impacting post-translational modifications such as protein acetylation and phosphorylation, as well as the transcription of tumor-associated genes. This, in turn, promotes tumor proliferation, invasion, and resistance to therapy (Lee et al., 2017; Lee et al., 2018). Based on this premise, the targeting of PFKP and its associated metabolic networks has emerged as a promising strategy for tumor metabolic therapy. Notably, AZD3965, an MCT1 inhibitor that targets lactate transport downstream of PFKP, has progressed to phase I/II clinical trials, while several small-molecule PFKP inhibitors have successfully undergone preclinical validation (Halford et al., 2021; Halford et al., 2023; Xu et al., 2025), These developments underscore the translational potential of PFKP in the context of precision oncology.

The research trajectory of phosphofructokinase platelet (PFKP) reflects the shifting paradigm in tumor metabolism research, which has transitioned from an emphasis on canonical glycolysis to a more integrated approach that encompasses coordinated metabolic and signaling regulation. PFKP, as a critical molecule with both enzymatic catalytic activity and non-metabolic regulatory functions, plays a significant role in driving tumor metabolic reprogramming and influencing tumor initiation and progression through various mechanisms, including subcellular translocation, epigenetic regulation, and remodeling of the tumor microenvironment, thereby exhibiting substantial translational potential. With the progressive elucidation of the regulatory network governing PFKP, there is increasing interest in investigating its potential as a diagnostic biomarker and a target for metabolic therapy. This review systematically synthesizes the latest research advancements concerning PFKP, focusing on its structural and biological functions, its mechanistic roles in tumorigenesis and tumor progression, its interactions with the tumor microenvironment, and its translational potential. The aim is to provide novel theoretical insights for PFKP-targeted therapy and precision cancer medicine (Figure 1).

**FIGURE 1:**
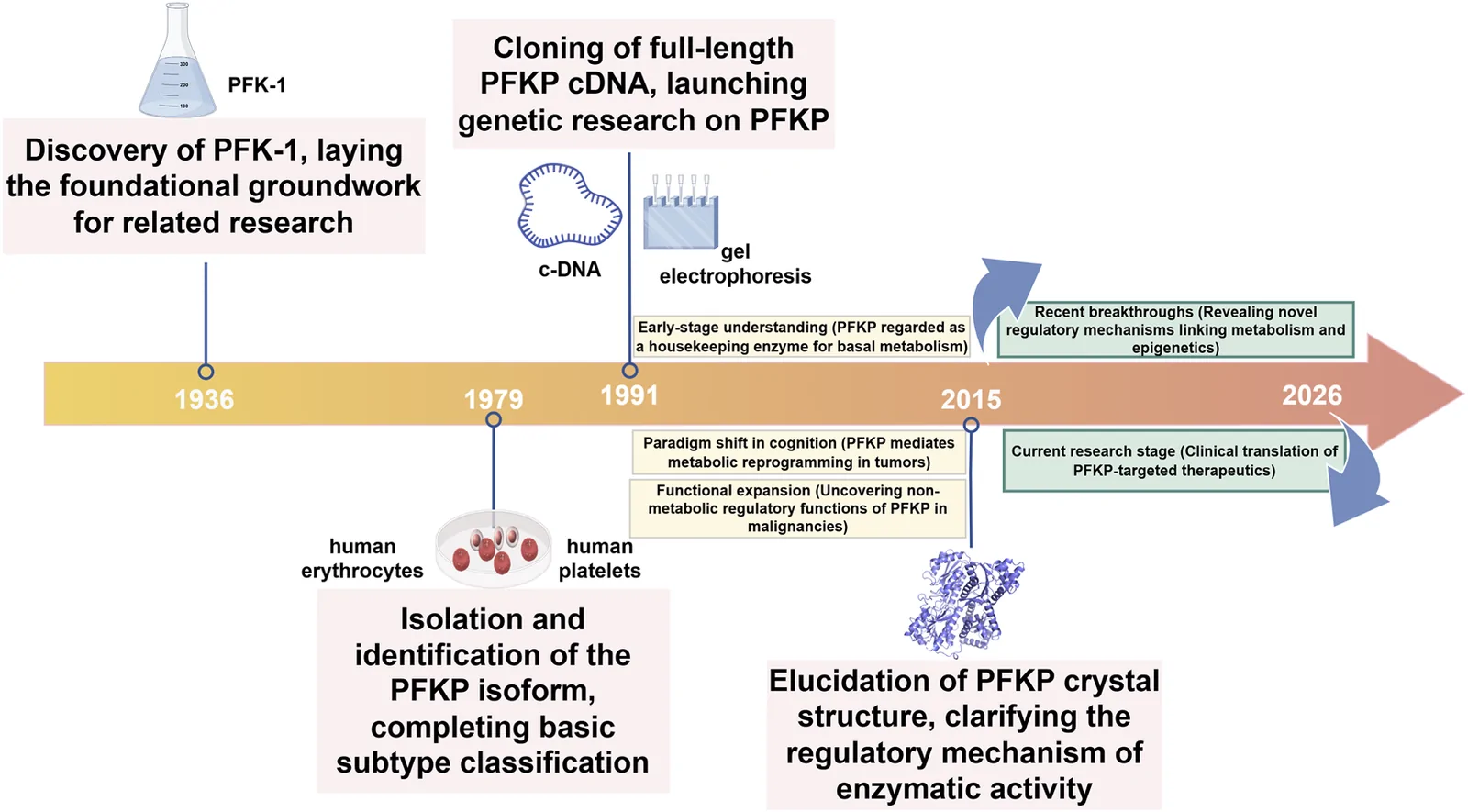
(By Figdraw): Research history of PFKP.

## 3 Subcellular localization and biological functions of PFKP

### 3.1 Biological functions of PFKP in the cytoplasm

Platelet-type phosphofructokinase (PFKP) performs a variety of biological functions within the cytoplasm. In addition to its established role in the regulation of glucose metabolism, PFKP is involved in a wide range of non-metabolic biological processes, such as kinase-mediated signaling, autophagy regulation, and cytoskeletal remodeling. To facilitate a clear organizational framework, the subsequent section is divided into two distinct modules: metabolic regulatory functions, as well as non-metabolic kinase and signaling activities.

#### 3.1.1 Metabolic regulatory functions of PFKP

PFKP serves as a fundamental regulator of glycolysis, playing a crucial role in sustaining cellular metabolic homeostasis by modulating glycolytic flux and influencing the levels of metabolic intermediates. From a metabolic perspective, the concurrent overexpression of PFKP and inhibition of aldolase B (ALDOB) can restore the cytoskeletal architecture of podocytes by modulating fructose-1,6-bisphosphate (FBP) levels and inhibiting the RhoA/ROCK1 signaling pathway (Zhang et al., 2022). Coactivator-associated arginine methyltransferase 1 (CARM1) facilitates the methylation of PPP1CA at the R23 residue, consequently enhancing the activity of phosphofructokinase platelet (PFKP) and promoting glycolytic flux (Zhang L. et al., 2023).

The metabolic functions of phosphofructokinase platelet (PFKP) are not exclusive to malignant tumors; rather, PFKP is involved in the initiation and progression of various non-neoplastic diseases. Investigating these conserved regulatory mechanisms is anticipated to offer new research avenues for elucidating the principles governing tumor evolution. For example, abnormal expression of phosphofructokinase platelet type (PFKP) has been observed in various disorders, including migraine, renal interstitial fibrosis, and pulmonary arterial hypertension (PAH). In these conditions, PFKP is involved in pathological processes such as neuroinflammation, metabolic reprogramming of renal tubular epithelial cells, and proliferation of vascular smooth muscle cells. In experimental models of migraine, PFKP expression is notably increased in the spinal dorsal horn, and the glycolytic inhibitor 2-deoxy-D-glucose (2-DG) has been demonstrated to effectively mitigate migraine-like symptoms (Qiu et al., 2023). In renal tubular epithelial cells, phosphofructokinase platelet (PFKP) influences the advancement of renal interstitial fibrosis through the regulation of glycolysis (Yang et al., 2023). In the context of pulmonary arterial hypertension, the activation of peroxisome proliferator-activated receptor gamma (PPARγ) in vascular smooth muscle cells (VSMCs) suppresses transforming growth factor beta 1 (TGFβ1)-induced mitochondrial activation and VSMC proliferation. This activation concurrently downregulates PFKP, thereby modulating cellular proliferation and glucose metabolism (Calvier et al., 2017). Importantly, the mechanistic insights derived from non-tumor models cannot be directly extrapolated to cancer research. The application of these non-neoplastic mechanisms to understand tumor progression remains speculative and necessitates further empirical validation within malignant contexts.

#### 3.1.2 Non-metabolic kinase and signaling regulatory functions of PFKP

Recent research has demonstrated that PFKP is implicated in a variety of non-glycolytic biological processes. In conditions of amino acid deprivation, PFKP acts as a protein kinase to regulate the phosphorylation of ATG4B, thereby facilitating autophagy (Li et al., 2021). It also catalyzes the serine phosphorylation of the developmental regulator Lin41 (Cao et al., 2023). Furthermore, PFKP is involved in the metabolic reprogramming of microsatellite instability (MSI) tumors (Li Q. et al., 2023). Mitochondrial phosphoenolpyruvate carboxykinase (PCK2) augments PFKP activity, thereby activating the AKT/extracellular signal-regulated kinase 1/2 (ERK1/2) signaling pathway, which promotes the osteogenic differentiation of mesenchymal stem cells (Lee et al., 2018). Under ethanol exposure, SIRT2 interacts with PFKP to decrease the phosphorylation of Atg4B and the activation of microtubule-associated protein 1 light chain 3B (LC3), thus inhibiting LC3-associated phagocytosis (LAP)(Zhou et al., 2018). In the context of stem cell differentiation regulation, mitochondrial phosphoenolpyruvate carboxykinase (PCK2) augments the enzymatic activity of PFKP and activates the AKT/ERK1/2 signaling pathway, thereby influencing the osteogenic differentiation of mesenchymal stem cells (MSCs)(Li et al., 2022).

Additionally, the non-metabolic roles of PFKP extend beyond malignant tumors, as PFKP is also involved in the initiation and progression of various non-neoplastic diseases. Investigating these conserved regulatory mechanisms is anticipated to provide new research avenues for elucidating the principles underlying tumor evolution. For example, in acute myeloid leukemia (AML), the lncRNA XIST/miR-142–5p/PFKP axis modulates PFKP expression, impacting cellular proliferation, cell cycle progression, and apoptosis (Jiang et al., 2024; Calvier et al., 2017; Luo et al., 2018). PFKP is also implicated in cardiac hypertrophy and the regulation of coronary endothelial cell function (Vigil-Garcia et al., 2021), with its inhibition shown to mitigate pathological cardiac hypertrophy (Zhang et al., 2024; Wu et al., 2024). Moreover, anti-human protein S antibodies have been found to bind directly to PFKP, activating ERK1/2 and inducing tissue factor expression, which underscores the significant role of PFKP in the pathogenesis and progression of cardiovascular diseases (Chen et al., 2014; Liao et al., 2009).

### 3.2 Biological functions of nuclear PFKP

PFKP is primarily localized in the cytoplasm where it performs glycolytic functions. However, under certain conditions, it translocates to the nucleus, where it exerts non-canonical regulatory effects that are independent of its enzymatic activity in metabolism. Recent research has revealed that nuclear-localized PFKP serves as a crucial nexus connecting metabolic reprogramming with transcriptional regulation. This finding is consistent with the recently proposed “moonlight function” theory of metabolic enzymes, which suggests that these enzymes can extend beyond their traditional metabolic roles to engage in transcriptional regulation, chromatin remodeling, and signal transduction within the nucleus.

Significantly, the majority of mechanistic insights into the nucleocytoplasmic shuttling and nuclear functions of PFKP have been primarily obtained from a limited number of studies focused on T-cell acute lymphoblastic leukemia (T-ALL). Consequently, experimental evidence pertaining to other tumor models remains scarce. In T-cell acute lymphoblastic leukemia (T-ALL), the Cyclin D3/CDK6 complex facilitates the nuclear import of phosphofructokinase platelet type (PFKP). Once localized in the nucleus, PFKP activates c-Myc-dependent transcription of CXCR4, thereby promoting the infiltration and invasion of leukemic cells. The expression level of nuclear PFKP is significantly correlated with the invasiveness and poor prognosis of T-cell malignancies, underscoring its potential utility in diagnostic and prognostic assessments (Broxmeyer, 2021; Gao et al., 2021). Furthermore, a marked nuclear accumulation of PFKP is observed in malignancies associated with diabetes, suggesting that its nuclear localization mediates the interplay between metabolic disorders and the initiation and progression of tumors (Liu et al., 2026a). Nevertheless, several critical questions remain incompletely resolved in existing literature, including the precise molecular machinery governing PFKP nuclear import and export, the functional relevance of its oligomeric state during subcellular translocation, essential post-translational modifications controlling its shuttling behavior, and additional direct experimental evidence verifying its catalytic-independent activities in the nucleus.

Collectively, the subcellular localization of PFKP significantly influences its functional diversity: cytoplasmic PFKP predominantly facilitates glycolytic metabolism, while intranuclear PFKP is primarily involved in gene transcription and signal regulation. Given the limited availability of data from non-T-ALL models, caution should be exercised when generalizing these mechanistic models to other cancer types. This transition of PFKP from a metabolic enzyme to an intranuclear regulatory factor enhances our comprehension of its biological roles and offers novel theoretical insights for precision therapies targeting PFKP (Figure 2).

**FIGURE 2:**
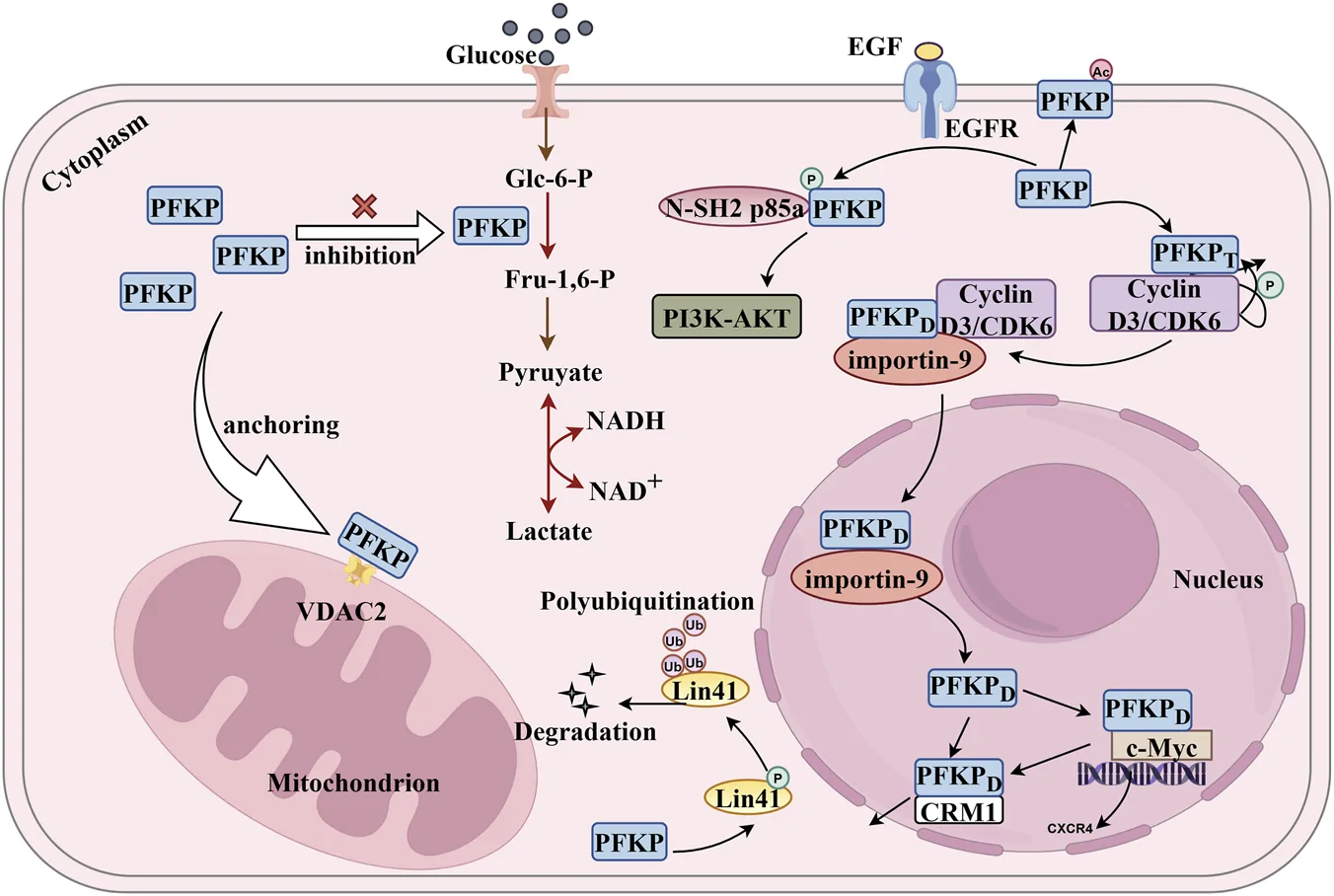
(By Figdraw): Subcellular localization and biological functions of PFKP.

## 4 PFKP-mediated metabolic and non-metabolic regulatory networks driving tumor progression

### 4.1 PFKP-driven tumor metabolic reprogramming

Metabolic reprogramming represents a crucial hallmark that confers tumor cells with the ability to maintain sustained proliferative capacity and adapt to environmental changes. Phosphofructokinase platelet type (PFKP), as the principal rate-limiting enzyme of the glycolytic pathway, is acknowledged as a pivotal nexus linking oncogenic signaling with metabolic remodeling. Recent studies have revealed that the functions of PFKP extend significantly beyond its traditional role as a glycolytic enzyme. It not only supports the Warburg effect by modulating glycolytic flux but also orchestrates glucose metabolism, fatty acid oxidation, and mitochondrial energy metabolism, thereby imparting substantial metabolic plasticity to tumor cells. In this context, metabolic plasticity denotes the ability of malignant cells to adaptively transition between various energy metabolic pathways in response to changing nutrient availability and microenvironmental pressures, thereby facilitating survival under metabolically restrictive conditions. Consequently, PFKP emerges as a key effector molecule in driving tumor metabolic reprogramming.

A substantial body of research currently demonstrates that PFKP-mediated metabolic reprogramming is characterized by upstream regulatory heterogeneity, yet it results in convergent metabolic outcomes. While diverse tumors utilize distinct oncogenic pathways to activate PFKP, they ultimately converge on the shared metabolic endpoint of augmented glycolysis. It should be noted that existing evidence varies widely in causal validity: clinical transcriptomic signatures and correlational expression analyses merely demonstrate statistical associations between elevated PFKP and malignant phenotypes, whereas transient overexpression or genetic silencing assays establish functional relevance but cannot fully distinguish indirect metabolic consequences from direct signaling effects (Fendt et al., 2020). For instance, in head and neck squamous cell carcinoma (HNSCC), elevated PFKP levels facilitate tumor progression and glycolytic metabolism through the AMOTL1/YAP/Hippo signaling pathway (Wang L. et al., 2026). In oral squamous cell carcinoma (OSCC), nutrient deprivation conditions lead to PFKP upregulation, which subsequently enhances glycolysis, autophagy, and epithelial–mesenchymal transition (EMT) (Chen et al., 2018). In thyroid cancer, P4HA2 (prolyl-4-hydroxylase subunit 2) promotes glycolysis via PFKP to support tumor growth (Sun et al., 2025). In breast cancer, both PITX1 and the MORC2–MAX–MYC transcriptional regulatory axis increase PFKP expression and glycolytic activity (Cho et al., 2020; Guddeti et al., 2023; Inaishi et al., 2022; Liu H. et al., 2026). The regulatory mechanisms of PFKP in tumors of the digestive system demonstrate bidirectional specificity. In gastric cancer, miR-186 targets and inhibits hypoxia-inducible factor HIF-1α, leading to the downregulation of PFKP expression and the subsequent inhibition of glycolysis, thereby suppressing cancer progression (Liu et al., 2016). Conversely, in hepatocellular carcinoma and pancreatic cancer, PFKP expression is upregulated through miR-520 and high-glucose–HIF-1α signaling pathways, respectively, facilitating tumor metabolic remodeling (Cheng et al., 2019; Park et al., 2013). Additionally, elevated PFKP expression is strongly correlated with increased glycolysis, cell proliferation, and malignant progression in cervical cancer, endometrial cancer, melanoma, and bladder cancer (Cengiz et al., 2024; Cengiz, 2024; Sun et al., 2016; Chen and Zhang, 2023).

Accumulating evidence indicates that the primary function of PFKP is to maintain the metabolic adaptability of tumor cells, rather than solely enhancing glycolysis. Under conditions of glucose deprivation, PFKP interacts with AMPK in non-small cell lung cancer, facilitating mitochondrial recruitment and long-chain fatty acid oxidation, thereby mitigating metabolic stress and improving tumor cell survival in nutrient-limited environments (Chen et al., 2022; Wang et al., 2021). Similarly, in intrahepatic cholangiocarcinoma, mutant IDH1 depends on PFKP to activate AMPK signaling, thereby increasing cellular resilience to energy stress (Fujiwara et al., 2019). Nevertheless, important exceptions have been reported. In pancreatic ductal adenocarcinoma (PDAC), tumor cells rely predominantly on MCT1-mediated lactate uptake and oxidative phosphorylation, accompanied by suppressed expression of glycolytic enzymes including PFKP(Shao et al., 2020). Such divergence likely arises from tumor subtype-specific nutrient preference, unique stromal metabolic crosstalk, and heterogeneous microenvironmental constraints rather than contradicting the core function of PFKP in shaping metabolic plasticity. These findings collectively suggest that the function of PFKP is modulated by the tumor microenvironment and nutrient availability. By integrating various oncogenic signals to coordinate distinct energy metabolic pathways, PFKP plays a crucial role not only in promoting glycolysis but, more importantly, in sustaining tumor metabolic plasticity.

### 4.2 Non-canonical signaling regulatory functions of PFKP

As research advances, the oncogenic implications of PFKP have extended beyond mere metabolic regulation. A growing body of evidence suggests that PFKP acts as a signaling regulatory molecule, engaging in various biological processes such as cell proliferation, invasion and metastasis, angiogenesis, cancer stemness, and immune evasion. In recent years, its non-canonical functions have emerged as a significant focus of PFKP-related research.

To avoid ambiguity, we establish explicit criteria for discriminating glycolysis-dependent effects from non-canonical functions of PFKP. Phenotypes that can be fully rescued by exogenous metabolites following PFKP perturbation are attributed to impaired glycolytic flux. In contrast, non-canonical signaling functions are defined as effects retained upon expression of catalytically inactive PFKP mutants independent of glycolytic enzymatic activity (Pan et al., 2021; Guo et al., 2025). Notably, most current studies rely solely on PFKP overexpression or silencing, which cannot reliably separate secondary metabolic outcomes from direct non-canonical signaling activity unless catalytic-dead mutant controls are included.

PFKP serves as a critical integrator of various oncogenic signaling pathways, thereby amplifying the malignant phenotypes of tumor cells. In the context of non-small cell lung cancer (NSCLC), PFKP upregulation, mediated by zinc finger protein 148 (ZNF148), modulates PD-L1 expression, which in turn enhances the proliferative, invasive, and immune evasion capabilities of tumor cells (Zhu et al., 2023). Additionally, PFKP interacts directly with the AXL-MET signaling axis, promoting lung cancer progression in a manner that is partially independent of its glycolytic functions (Zhao et al., 2024; Sun et al., 2024). This work represents relatively robust evidence for non-canonical activity, as researchers partially decoupled phenotypes from glycolytic capacity. By contrast, many other observations described below are derived from correlational analyses or simple knockdown/overexpression systems, leaving open the possibility that phenotypes arise indirectly from altered glucose metabolism. In breast cancer, PFKP contributes to increased VEGF expression, thereby facilitating tumor angiogenesis (Cho et al., 2020; JONG-HOLEE, 2023). In colorectal cancer (CRC), PFKP activates the NF-κB signaling pathway, leading to the upregulation of GLUT1 and HK2, and establishing a positive feedback loop between metabolic processes and signal transduction, which further accelerates tumor progression (Deng et al., 2023).

In addition to its role in regulating cellular signaling, PFKP is implicated in the maintenance of cancer stemness, as well as in the processes of invasion and metastasis. Specifically, in ovarian cancer, PFKP promotes tumor metastasis, anchorage-independent growth, and the preservation of stemness through the α5 integrin/ERK/MMP9 signaling pathway (Zhang R. et al., 2023). In the context of hepatocellular carcinoma (HCC), the silencing of PFKP significantly reduces the expression of stemness markers and diminishes colony-forming capacity, highlighting its potential as a therapeutic target for HCC(Sha et al., 2021). Moreover, in colorectal cancer (CRC), PFKP appears to influence cell cycle progression, thereby supporting the sustained proliferation and invasion of cancer cells (Lu, 2023; Lu et al., 2023). Collectively, PFKP extends beyond its traditional role as a metabolic enzyme, functioning as a pivotal link between tumor metabolic reprogramming and signal transduction networks. Its non-canonical activities significantly expand the understanding of PFKP’s biological functions within cancer research. We also acknowledge that further experiments utilizing catalytic-deficient PFKP mutants are required across most tumor models to rigorously validate these proposed non-canonical functions and exclude indirect glycolysis-mediated effects (Figure 3).

**FIGURE 3:**
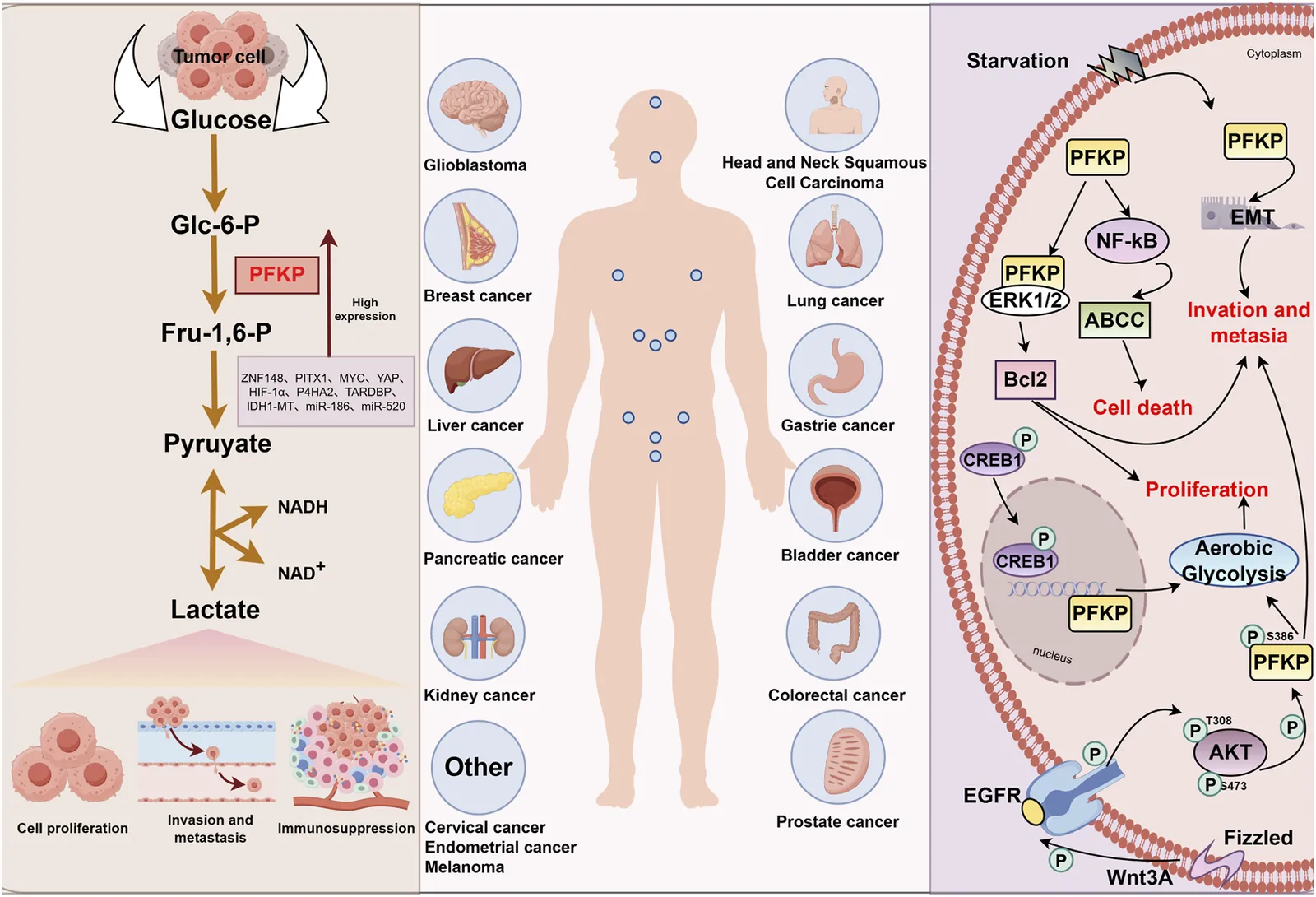
(By Figdraw): Metabolic and non-metabolic mechanisms of PFKP in various cancers.

### 4.3 PFKP remodels the tumor immune microenvironment

Tumor immune evasion constitutes a fundamental mechanism by which neoplasms circumvent immune surveillance and advance malignantly. This process is primarily facilitated through various pathways, including the activation of the PD-L1/PD-1 immune checkpoint, immune dysregulation within the tumor microenvironment, and compromised antigen presentation due to the downregulation of MHC molecules. These pathways represent critical research targets in contemporary cancer immunotherapy (Korotaeva et al., 2023; Liu et al., 2022; Mo et al., 2022).

As a fundamental regulator of metabolism, phosphofructokinase platelet (PFKP) has been demonstrated to play a role in the remodeling of the tumor immune microenvironment. In cases of clear cell renal cell carcinoma, there is a significant upregulation of PFKP expression. This overexpression not only facilitates tumor progression but also shows a strong association with the infiltration of B cells, CD8^+^ T cells, CD4^+^ T cells, macrophages, neutrophils, and dendritic cells. These findings suggest that PFKP may influence the recruitment of immune cells and the composition of the immune microenvironment by modulating metabolic pathways (Wang et al., 2016; Liu et al., 2021). In glioblastoma (GBM), the phosphorylation of PFKP at tyrosine 64 is strongly positively correlated with the expression of PD-L1, indicating that post-translational modifications of PFKP may play a crucial role in regulating tumor immune evasion (Wang D. et al., 2022). Research on hepatocellular carcinoma (HCC) has corroborated the pivotal role of PFKP in modulating responses to tumor immunotherapy (Yao et al., 2024). Furthermore, aberrantly elevated expression of PFKP alters the “immune desert” microenvironment, characterized by insufficient CD8^+^ T cell infiltration in lung adenocarcinoma (LUAD), thereby diminishing the therapeutic effectiveness of radiotherapy and anti-PD-L1 treatment (Han et al., 2024). In the context of uveal melanoma, interleukin-6 (IL-6) markedly upregulates glycolytic rate-limiting enzymes, including PFKP, while concurrently suppressing the acetyl-CoA synthetic pathway, collectively promoting immune evasion through metabolic reprogramming (Gong et al., 2024). In head and neck squamous cell carcinoma (HNSCC), the combination of ganetespib and radiotherapy counteracts the radiotherapy-induced upregulation of PFKP and PKM2, thereby enhancing T cell-mediated anti-tumor immune responses (Chen F. et al., 2024). Accumulated evidence from numerous studies indicates that PFKP exerts extensive immunomodulatory effects across a variety of tumors. Its overexpression is closely associated with decreased sensitivity to anti-tumor agents, positioning it as a pivotal molecule in mediating therapeutic resistance and reshaping the immune microenvironment (Peng et al., 2023). In contrast to its immune-evasive role in most solid tumors, PFKP diminishes the immune evasion capacity of leukemic stem cells by facilitating MYC protein degradation, thereby exerting a positive regulatory influence on anti-tumor immunity (Xu et al., 2024). Collectively, these findings illustrate that PFKP not only reconfigures tumor cell metabolism but also contributes to the establishment of an immunosuppressive tumor microenvironment by modulating immune cell infiltration and immune checkpoint expression. This lays a novel theoretical foundation for PFKP-targeted combination immunotherapy.

### 4.4 PFKP promotes therapeutic resistance in tumors

Emerging evidence indicates that the dysregulated activation of PFKP plays a critical role in tumor initiation and progression, while also contributing to the development of chemoresistance through various mechanisms. It is important to distinguish two major forms of drug resistance: intrinsic resistance, defined as innate insensitivity to chemotherapy present prior to drug exposure, and acquired resistance, which emerges dynamically after sustained drug treatment (Holohan et al., 2013; Lei et al., 2023). In addition, experimental evidence varies in translational reliability, including *in vitro* cell line observations, *in vivo* animal model data, and clinical correlational findings derived from patient specimens (Sajjad et al., 2021). At present, most published studies do not explicitly discriminate whether PFKP mediates intrinsic or acquired chemoresistance. These mechanisms include metabolic reprogramming, the maintenance of cancer stem cell properties, and the activation of drug-resistance signaling pathways. In the context of non-small cell lung cancer (NSCLC), elevated PFKP expression is significantly correlated with chemoresistance in patients; this observation is derived from clinical correlational analysis. PFKP facilitates the activation of the NF-κB signaling pathway, leading to the upregulation of ABCC2 expression and subsequent induction of cisplatin resistance (Wang et al., 2023). In breast cancer, PFKP enhances glycolysis and increases the population of CD133^+^ cancer stem-like cells, thereby imparting chemoresistance to tumor cells (Fang et al., 2026). Furthermore, in cervical cancer (CC) and endometrial cancer, the overexpression of PFKP enhances tumor resistance to drugs through the activation of the p44/42 MAPK signaling pathway (Pires et al., 2017). Notably, these available evidences are limited to cell line-based research. In addition, other studies integrate *in vitro* cellular experiments and *in vivo* animal models for relevant validation. In colorectal cancer, the upregulation of glycolysis mediated by PFKP contributes to resistance against 5-fluorouracil, suggesting that PFKP could be a novel therapeutic target for overcoming drug resistance in colorectal cancer (Deng et al., 2023). Similarly, in pancreatic ductal adenocarcinoma (PDAC), the c-Myc–USP14–PFKP regulatory axis promotes tumor progression and resistance to gemcitabine (Wang P. et al., 2026). Current research collectively indicates that targeting PFKP constitutes a promising strategy for enhancing the therapeutic sensitivity of malignant tumors.

## 5 Post-translational modifications and protein-protein interactions regulate cancer progression

Protein post-translational modifications (PTMs) play a pivotal role in reversibly modulating protein conformation, enzymatic activity, subcellular localization, and protein stability, thereby intricately regulating a wide array of cellular physiological processes. The dysregulation of PTM homeostasis is a fundamental molecular characteristic that contributes to the initiation and progression of tumors (Xu et al., 2014; Wang et al., 2024). Protein-protein interactions (PPIs) are essential for intracellular signal transduction, substance metabolism, and gene transcription. The disruption of the dynamic equilibrium between the assembly and disassembly of protein complexes significantly contributes to the advancement of malignant tumors (Su et al., 2021). PTMs function as molecular switches that dynamically adjust PPIs, and the aberrant collaborative regulatory networks formed by these interactions are critical drivers of cancer development.

### 5.1 Post-translational modifications regulate PFKP function to promote cancer progression

Post-translational modifications (PTMs) are crucial regulatory mechanisms that influence protein function. By modifying protein conformation, enzymatic activity, stability, and subcellular localization, PTMs facilitate precise control over cellular metabolism and signal transduction pathways. Recent research has elucidated that phosphofructokinase, platelet type (PFKP), a key rate-limiting enzyme in glycolysis, is subject to dynamic modulation through various post-translational modifications, including phosphorylation, acetylation, ubiquitination, and lactylation. These modifications not only impact the catalytic activity of PFKP but also influence its protein stability, subcellular localization, and non-canonical biological functions, collectively contributing to tumor initiation and progression.

To date, phosphorylation represents the most extensively studied post-translational modification of phosphofructokinase platelet type (PFKP). Upon Wnt3A-induced reactivation of epidermal growth factor receptor (EGFR), the phosphoinositide 3-kinase (PI3K)/AKT signaling cascade induces phosphorylation of PFKP at serine 386, leading to increased PFKP abundance, enhanced glycolysis, and promotion of tumor cell proliferation, colony formation, and migration (Jeon et al., 2021). In glioblastoma (GBM), a positive feedback regulatory loop is observed: the phosphorylation level of PFKP at tyrosine 64 is positively correlated with hypoxia-inducible factor 1-alpha (HIF-1α), serine 552-phosphorylated β-catenin, and vascular endothelial growth factor (VEGF) expression. VEGF, in turn, augments PFKP expression and enzymatic activity in a manner dependent on PFKP serine 386 phosphorylation, thereby accelerating aerobic glycolysis and GBM cell proliferation (Lim et al., 2022; Lee et al., 2020). Concurrently, AKT directly interacts with and phosphorylates PFKP at serine 386, inhibiting the interaction between the E3 ubiquitin ligase TRIM21 and PFKP, suppressing TRIM21-mediated polyubiquitination and degradation of PFKP, maintaining PFKP protein stability, and further promoting aerobic glycolysis, cell proliferation, and intracranial tumor growth (Lee et al., 2017). Furthermore, analyses of clinical GBM specimens have verified that Tyr64 phosphorylation of PFKP is positively associated with PD-L1 expression, indicating that this modification bears important clinical implications for immune evasion in glioma (Wang S. et al., 2022).

In addition to its role in regulating tumor metabolism and malignant phenotypes, PFKP phosphorylation is also involved in DNA damage repair, modulation of autophagy, and the regulation of tumor sensitivity to radiotherapy and chemotherapy. The ATM kinase phosphorylates PFKP at the T278 residue, facilitating its translocation to the nucleus. This translocation promotes the recruitment of casein kinase 2 (CK2) and enhances the interaction between RAD51 and BRCA2, thereby increasing the efficiency of homologous recombination (HR) repair. Concurrent targeting of the glycolytic and HR repair pathways can significantly enhance tumor radiosensitivity (Liu H. et al., 2026). PFKP phosphorylation plays critical roles not only in tumor progression but also in the pathogenesis of multiple non-neoplastic diseases. Elucidating its conserved regulatory rules can provide innovative strategies for tumor research. Some studies have demonstrated that phosphorylation of PFKP at S386 is a critical requirement for the activation of the autophagy-related protein Atg4B and the regulation of cellular autophagy (Li et al., 2021). Additionally, CaMK4 phosphorylates and activates PFKP, thereby accelerating aerobic glycolysis in cells and broadening the scope of PFKP’s functions (Scherlinger et al., 2022).

In addition to phosphorylation, acetylation represents a crucial mechanism for regulating PFKP function. In the context of brain tumors, KAT5-mediated acetylation of PFKP at the K395 residue facilitates its translocation to the cell membrane, where it subsequently undergoes EGFR-induced phosphorylation at Y64. This sequential process ultimately activates the PI3K/AKT signaling pathway, enhances glycolysis, and drives brain tumorigenesis (Lee et al., 2018). Furthermore, the acetylation of PFKP at K395 is intricately associated with its ability to function as a protein kinase and activate ATG4B, indicating that acetylation not only influences the catalytic activity of this metabolic enzyme but also contributes to the non-canonical signaling regulatory roles of PFKP(Gandhirajan et al., 2023).

In recent years, lactylation has emerged as a significant post-translational modification, attracting considerable scholarly attention. Research has demonstrated that lactylation of phosphofructokinase platelet (PFKP) displays notable tissue and disease specificity. Specifically, in ovarian cancer, lactylation of PFKP at lysine 392 (K392) enhances its enzymatic activity, thereby accelerating glycolysis and tumor progression through the repression of PTEN (Mi et al., 2024). Conversely, in a myocardial ischemia model, lactylation at lysine 688 (K688) increases glycolytic flux and confers cardioprotective effects (Liu et al., 2026a). Interestingly, in colorectal cancer, lactylation of PFKP at K688 reduces its catalytic activity, suggesting that PFKP lactylation may regulate glycolysis and lactate production via a negative feedback mechanism to maintain cellular metabolic homeostasis (Cheng et al., 2024). The contradictory functional outcomes resulting from PFKP K688 lactylation underscore the context-dependent nature of this modification. These divergent effects are likely attributable to variations in cell type, intracellular metabolite environments, concurrent post-translational modifications, and associated interacting protein networks. However, the upstream regulatory enzymes and downstream molecular events that govern this tissue-specific functional switch remain largely uncharacterized. Further research is necessary to elucidate the precise mechanisms that determine the divergent biological consequences of K688 lactylation.

In addition to directly modifying enzymatic activity, ubiquitination predominantly regulates the stability of the PFKP protein. In the context of breast cancer, USP5 and RNF123 collaboratively modulate the deubiquitination and stability of PFKP, thereby maintaining glycolytic activity in tumor cells and promoting tumor progression (Chen et al., 2024a; Peng et al., 2024). Conversely, HRD1 interacts with cytoplasmic PFKP in breast cancer cells, inducing its ubiquitination and subsequent degradation, which in turn suppresses tumor growth and metastasis (Fan et al., 2021). Furthermore, phosphorylation of PFKP at serine 386 by AKT disrupts its interaction with TRIM21, preventing ubiquitin-dependent degradation of PFKP (Lee et al., 2017). Collectively, these studies illustrate the complex interplay among various post-translational modifications, which collectively influence the abundance and functional state of PFKP.

In conclusion, a variety of post-translational modifications collectively form a regulatory network that governs the function of phosphofructokinase platelet (PFKP). These modifications influence PFKP by modulating its enzymatic activity, protein stability, subcellular localization, and non-canonical roles, thereby driving critical pathological processes such as tumor metabolic reprogramming, DNA damage repair, autophagy, and therapeutic resistance.

### 5.2 Protein–protein interaction networks endow PFKP with non-canonical oncogenic functions

In addition to post-translational modifications, phosphofructokinase platelet type (PFKP) engages in dynamic protein–protein interaction (PPI) networks with a diverse array of proteins, thereby transcending its conventional role as a metabolic enzyme. In this study, we concentrate solely on experimentally validated direct physical binding interactions between PFKP and its partner proteins, differentiating these physical interactions from indirect pathway correlations or reciprocal regulatory associations. It plays a significant role in cellular signal transduction, metabolic adaptation, and the remodeling of the tumor microenvironment. Increasing evidence suggests that PFKP functions not only as an effector molecule in glycolysis but also as a pivotal hub that interconnects metabolic regulation with oncogenic signaling networks. PFKP forms complexes with various proteins to influence tumor progression. In head and neck squamous cell carcinoma (HNSCC), PFKP directly physically interacts with AMOTL1, inhibiting its ubiquitination and subsequent degradation. This interaction remodels tumor metabolism and promotes epithelial-mesenchymal transition (EMT) in HNSCC through the AMOTL1/YAP/Hippo signaling axis (Wang L. et al., 2026). In the context of non-small cell lung cancer (NSCLC), PFKP physically associates with AMPK, thereby enhancing cancer cell survival under conditions of glucose deprivation and bolstering the metabolic adaptability of tumor cells (Chen et al., 2022). Additionally, PFKP directly binds to AXL, facilitating its phosphorylation at Y779, which activates the AXL signaling cascade and further enhances the invasive and metastatic potential of tumor cells (Zhao et al., 2024; Sun et al., 2024).

Phosphofructokinase, platelet type (PFKP), plays a crucial role in maintaining cancer stemness and facilitating metabolic remodeling through its physical interactions key regulatory proteins. In the context of glioblastoma (GBM), PFKP not only physically interacts with vascular endothelial growth factor (VEGF) to enhance GBM proliferation but also forms a complex with the mitochondrial membrane protein voltage-dependent anion channel 2 (VDAC2) to regulate glucose metabolism and the phenotypic reprogramming of glioma stem cells (Lim et al., 2022; Zhou et al., 2018). Conversely, in breast cancer, the E3 ubiquitin ligase HRD1 physically interacts with PFKP, leading to its ubiquitination and subsequent degradation, thereby inhibiting the proliferation and metastasis of breast cancer cells (Fan et al., 2021). Furthermore, the environmental contaminant tris(2-chloroethyl) phosphate (TCEP) enhances the physical interaction between O-GlcNAc transferase (OGT) and PFKP, resulting in reduced PFKP enzymatic activity. This observation suggests that exogenous environmental factors may influence cellular metabolism by altering the PFKP interactome (Li S. et al., 2025).

In summary, the PFKP protein interactome demonstrates significant dynamism and context dependence, characterized by distinct interacting proteins that cooperatively regulate PFKP’s stability, metabolic activity, and signal transduction capacity. We emphasize that all protein partnerships listed above refer to confirmed physical interactions rather than indirect pathway-level regulation. Post-translational modifications play a crucial role in determining PFKP activation, while protein-protein interactions further influence the specific biological functions mediated by PFKP. These two regulatory layers are intricately linked, collectively conferring upon PFKP non-canonical oncogenic functions beyond metabolic regulation. This establishes PFKP as a critical molecular hub that interconnects glycolysis, signal transduction, cancer stemness, immune evasion, and therapeutic resistance (Figure 4).

**FIGURE 4:**
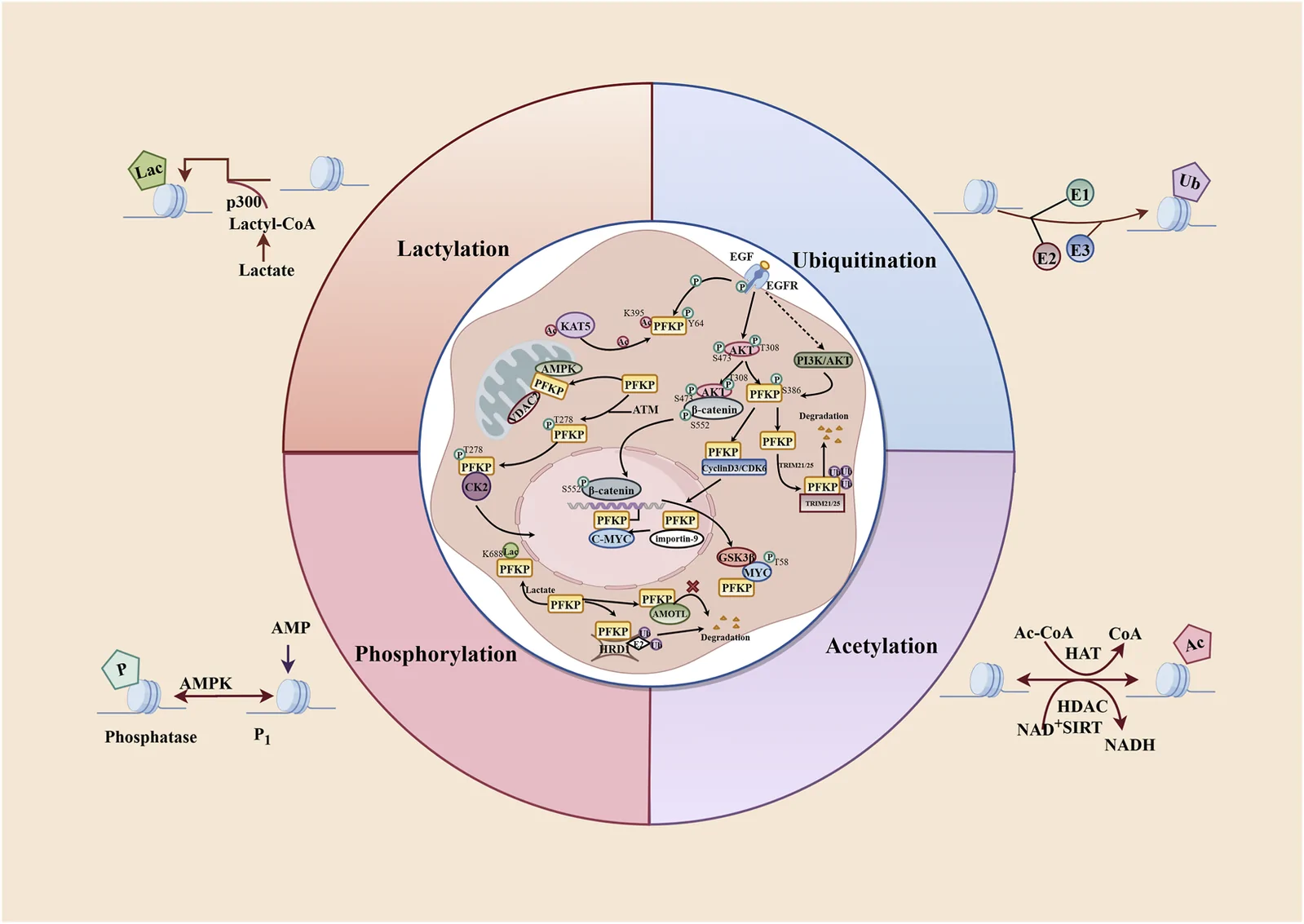
(By Figdraw): Modification mechanisms and interacting proteins of PFKP.

## 6 Regulatory network of PFKP expression drives tumor initiation and progression

To date, numerous transcription factors have been confirmed to directly regulate the transcription of PFKP. Specifically, c-Myc binds to the PFKP promoter, thereby facilitating its transcription, while PFKP, in turn, stabilizes the c-Myc protein through ERK signaling. This interaction establishes a positive feedback loop that enhances oncogenic metabolic reprogramming (Liu et al., 2024). In the context of breast cancer, KLF4 interacts directly with the PFKP promoter, activating its expression and thereby augmenting glycolysis and the proliferative capacity of tumor cells (Moon et al., 2011). Additionally, TGF-β1 upregulates PFKP via the SMAD3-SP1 transcriptional complex, contributing to renal fibrosis and aberrant metabolic remodeling (Yang et al., 2023). Collectively, these studies suggest that diverse oncogenic signaling pathways can activate PFKP transcription, thereby synergistically promoting tumor metabolic reprogramming.

In addition to transcriptional regulation, PFKP is subject to modulation through epigenetic modifications and its non-canonical functions within the nucleus. Comprehensive pan-cancer analyses have demonstrated a strong correlation between elevated PFKP expression and hypomethylation of its promoter, suggesting that aberrant DNA methylation is a critical mechanism underpinning sustained PFKP activation ((Peng et al., 2023). Moreover, in T-cell acute lymphoblastic leukemia (T-ALL), nuclear PFKP promotes c-Myc-dependent CXCR4 expression, while c-Myc, in turn, enhances the nuclear translocation of PFKP. This reciprocal interaction establishes a positive feedback loop that facilitates leukemic cell invasion (Gao et al., 2021). These complex, multi-layered regulatory networks work synergistically to maintain consistently elevated levels of PFKP in tumor cells, thereby facilitating tumor initiation and malignant progression. Importantly, nuclear PFKP functions as a scaffold protein that recruits CK2, promotes phosphorylation at the T13 residue, enhances the assembly of the RAD51-BRCA2 complex, increases the efficiency of homologous recombination (HR) repair, mitigates DNA damage, and consequently imparts radioresistance to tumor cells (Liu et al., 2026b). In response to EGF stimulation, CaMKK2 accumulates in the nucleus to regulate PFKP transcription, thereby influencing cancer cell metabolism and proliferation (Dai et al., 2022). Collectively, PFKP expression is regulated through a multi-tiered framework that includes transcriptional control, epigenetic modifications, and intranuclear regulatory networks. Its persistent overexpression not only supports tumor metabolic reprogramming but also enhances DNA damage repair and promotes malignant tumor progression.

## 7 Potential clinical application value of PFKP

With ongoing advancements in tumor metabolism research, PFKP has emerged as an important molecule governing tumor metabolic reprogramming, and exhibits promising potential for future clinical translation rather than established clinical utility. A multitude of clinical studies have shown that elevated PFKP expression is frequently associated with heightened tumor invasiveness, diminished patient survival rates, and suboptimal therapeutic responses. These findings suggest that PFKP may serve as a candidate metabolic biomarker, yet robust prospective clinical validation remains lacking for its routine application in tumor diagnosis, prognostic evaluation, and prediction of therapeutic efficacy.

Currently, elevated expression of PFKP is significantly correlated with reduced overall survival (OS), decreased disease-free survival (DFS), lymph node metastasis, and increased tumor malignancy across various solid tumors, including non-small cell lung cancer, head and neck squamous cell carcinoma, breast cancer, ovarian cancer, cervical cancer, and colorectal cancer (Bolanos-Suarez et al., 2023; Shen et al., 2020; Li et al., 2023; Wang P. et al., 2026; Lu, 2023; Umar et al., 2020; Lu et al., 2023; Bolaños-Suárez et al., 2022; Liu et al., 2024). Specifically, in head and neck squamous cell carcinoma, the ncRNA XIST/miR-29b-3p/COL3A1 axis promotes tumor progression by modulating PFKP-mediated central carbon metabolism, which is strongly associated with poor patient prognosis (Li et al., 2023; Wang P. et al., 2026; Liu et al., 2024). In colorectal cancer, multivariate Cox regression analyses further substantiate that elevated PFKP expression is an independent adverse prognostic factor for patients (Lu, 2023; Lu et al., 2023). Notably, such conclusions await verification in large-scale prospective clinical trials. Moreover, pan-cancer big data analyses have confirmed that PFKP overexpression constitutes a common independent prognostic risk factor across a range of malignant tumors (Ling et al., 2024).

The clinical significance of PFKP demonstrates notable tissue specificity across various tumor types. For example, PFKP provides substantial diagnostic and prognostic predictive value in clear cell renal cell carcinoma (KIRC) (Zhao et al., 2025). Conversely, increased PFKP expression is associated with favorable clinical outcomes in renal cell carcinoma (RCC) (Yuan et al., 2023). This observation suggests that the biological functions of PFKP may be influenced by tissue-specific metabolic landscapes and tumor microenvironments. Current evidence originates from retrospective analyses, bioinformatic predictions and preclinical investigations. Owing to inadequate clinical validation, PFKP is not ready for clinical use as a routine diagnostic and prognostic biomarker. Additional multi-omics studies are needed to unravel the functional heterogeneity of PFKP in different tumor microenvironments and facilitate the exploration of PFKP-targeted precision therapy and diagnosis.

## 8 Progress in PFKP-Targeted intervention strategies and drug development

As the crucial role of PFKP in tumor metabolic reprogramming continues to be elucidated, targeted intervention against PFKP has gradually become an important research direction in tumor metabolic therapy. At present, intervention strategies centered on PFKP have expanded from direct inhibition of its enzymatic activity to multiple regulatory levels, including transcriptional regulation, post-translational modification, protein stability control and remodeling of signaling networks. Natural active ingredients, small-molecule compounds and nucleic acid drugs all exhibit encouraging preclinical potential within such strategies.

Currently, a variety of natural active compounds exhibit anti-tumor properties by directly or indirectly inhibiting PFKP-mediated glycolysis. Isoliquiritigenin specifically targets the PITX1-PFKP axis to suppress glycolysis and impede the progression of breast cancer (Liu et al., 2026a). Suramin inhibits cervical carcinogenesis by obstructing PFKP-driven glycolytic signaling (Shi et al., 2026). Quercetin reduces PFKP mRNA and protein levels by inhibiting the PFKP-LDHA axis, thus suppressing cancer cell migration (Chen et al., 2024b; Umar et al., 2020). Triptolide disrupts GATA4-mediated glycolysis by decreasing PFKP protein abundance (Zhang L. et al., 2021). Ganetespib interferes with glycolysis in head and neck squamous cell carcinoma cells by inhibiting both the transcription and post-translational expression of PFKP(Chen et al., 2024a).

In addition to the direct inhibition of PFKP expression or its enzymatic activity, an increasing volume of research has begun to concentrate on the stability of the PFKP protein and its upstream regulatory networks. Both TRIM25 and TRIM21 promote the ubiquitination and subsequent proteasomal degradation of PFKP. Notably, CMBL enhances the interaction between TRIM25 and PFKP, thereby expediting the proteasome-mediated degradation of PFKP in colorectal cancer cells (Huang et al., 2023; Li Z. et al., 2025; Wen et al., 2024). Concurrently, the suppression of HOTAIRM1 inhibits glycolysis via the Wnt/β-catenin/PFKP axis, thereby increasing the sensitivity of acute myeloid leukemia cells to cytarabine (Chen et al., 2020). R-2-hydroxyglutarate (R-2HG) impedes the post-transcriptional upregulation of PFKP through the FTO/m^6^A/YTHDF2 pathway (Qing et al., 2021). Hyperbaric oxygen (HBO) therapy mitigates the Warburg effect and epithelial-mesenchymal transition (EMT) in non-small cell lung cancer by inhibiting the HIF-1α/PFKP axis (Zhang Y. et al., 2021). Moreover, alteration of the Asp-Arg allosteric regulatory site in PFKP significantly reduces its catalytic function, providing a novel theoretical basis for the development of PFKP allosteric inhibitors (Voronkova et al., 2023).

Despite its potential, PFKP is not universally recognized as a therapeutic inhibitory target due to its highly specific biological functions that vary across different tissues and diseases. For example, the inhibition of SHP-1 leads to the upregulation of PFKP expression via the AKT/β-catenin pathway, thereby enhancing energy metabolism in leukemic stem cells and increasing their susceptibility to chemotherapeutic agents (Xu et al., 2024). Conversely, compounds such as meldonium, isorhamnetin, Gen-miR-5, Xuesaitong injection, linefloxacin, Sal-miR-58, and indoxyl sulfate also influence PFKP expression or activity. However, their therapeutic applications are primarily directed towards non-malignant conditions, including cardiovascular diseases, renal fibrosis, and inflammation (Pant et al., 2025; Wang D. et al., 2022; Ge et al., 2025; Su et al., 2026; Jeong et al., 2024; Yang et al., 2023; Qin et al., 2020). This suggests that PFKP functions as a central metabolic regulatory hub, exerting significant biological effects across a wide range of diseases.

In summary, contemporary intervention strategies aimed at targeting PFKP have progressively evolved beyond traditional enzymatic activity inhibition to encompass a broader spectrum of approaches, including transcriptional regulation, post-translational modification, protein stability control, and metabolic network remodeling. However, the majority of agents targeting PFKP are still in the basic research or preclinical stages, necessitating further validation of their selectivity, pharmacokinetic profiles, and clinical safety. Looking ahead, the development of highly selective PFKP inhibitors, in conjunction with combinatorial therapies such as immunotherapy, radiotherapy, and other metabolism-targeted strategies, is anticipated to expedite the clinical translation of PFKP-targeted treatments (see Table 1).

**TABLE 1:** Summary of PFKP modulators, their mechanisms and associated diseases.

Category	Molecule Name	Mechanism and Description	Related Diseases/Tumor Types	References
Direct PFKP inhibitors	Ganetespib	Inhibits both transcription and post-translational expression of PFKP	Head and neck squamous cell carcinoma	Chen F et al., 2024
Direct PFKP inhibitors	Quercetin	Reduces mRNA and protein levels of PFKP, suppressing cancer cell migration	Multiple cancers	Chen F et al., 2024; Umar et al., 2020
Indirect regulatory molecules	Isoliquiritigenin	Targets the PITX1–PFKP axis to inhibit glycolysis	Breast cancer	Liu H. et al., 2026
Indirect regulatory molecules	Suramin	Blocks PFKP-driven glycolytic signaling	Cervical cancer	Shi et al., 2026
Indirect regulatory molecules	Triptolide	Decreases PFKP protein abundance and disrupts GATA4-mediated glycolysis	–	Zhang L. et al., 2021
Indirect regulatory molecules	R-2-hydroxyglutarate (R-2HG)	Suppresses post-transcriptional upregulation of PFKP via the FTO/m^6^A/YTHDF2 pathway	–	Qing et al., 2021
Indirect regulatory molecules	Hyperbaric oxygen (HBO)	Inhibits the HIF-1α/PFKP axis and alleviates the Warburg effect and EMT	Non-small cell lung cancer	Zhang L. et al., 2021
Upstream regulatory factors	TRIM25	E3 ubiquitin ligase promoting ubiquitination and proteasomal degradation of PFKP	Colorectal cancer	Huang et al., 2023; Wen et al., 2024
Upstream regulatory factors	TRIM21	E3 ubiquitin ligase promoting ubiquitination and proteasomal degradation of PFKP	–	Li S. et al., 2025
Upstream regulatory factors	CMBL	Strengthens TRIM25–PFKP interaction to accelerate PFKP proteasomal degradation	Colorectal cancer	Wen et al., 2024
Upstream regulatory factors	HOTAIRM1	lncRNA regulating glycolysis through the Wnt/β-catenin/PFKP axis	Acute myeloid leukemia	Chen et al., 2020
Upstream regulatory factors	PITX1	Transcription factor acting as a positive upstream regulator of PFKP	Breast cancer	Liu H. et al., 2026
Upstream regulatory factors	GATA4	Transcription factor mediating PFKP-related glycolytic programs	–	Zhang L. et al., 2021
Upstream regulatory factors	HIF-1α	Transcription factor that upregulates PFKP under hypoxia	Non-small cell lung cancer	Zhang L. et al., 2021
Upstream regulatory factors	SHP-1/AKT/β-catenin pathway	SHP-1 inhibition upregulates PFKP via AKT/β-catenin signaling; increased PFKP enhances chemosensitivity	Leukemic stem cells	Xu et al., 2024
Downstream metabolic targets	LDHA	Functions in the PFKP–LDHA axis; PFKP modulates LDHA to alter glycolytic flux	Migratory cancer cells	Chen F. et al., 2024; Umar et al., 2020
Downstream metabolic targets	Glycolysis (Warburg effect)	PFKP serves as the rate-limiting enzyme governing overall glycolytic activity	Multiple malignancies	Multiple references
Downstream metabolic targets	EMT (epithelial–mesenchymal transition)	PFKP-dependent glycolysis facilitates EMT and tumor metastasis	Non-small cell lung cancer	Zhang L. et al., 2021
Compounds studied only in non-neoplastic diseases	Meldonium	Modulates PFKP expression or activity	Cardiovascular diseases	Pant et al., 2025
Compounds studied only in non-neoplastic diseases	Isorhamnetin	Modulates PFKP expression or activity	–	Wang S. et al., 2022
Compounds studied only in non-neoplastic diseases	Gen-miR-5	Modulates PFKP expression or activity	–	Ge et al., 2025
Compounds studied only in non-neoplastic diseases	Xuesaitong injection	Modulates PFKP expression or activity	–	Su et al., 2026
Compounds studied only in non-neoplastic diseases	Linefloxacin	Modulates PFKP expression or activity	–	Jeong et al., 2024
Compounds studied only in non-neoplastic diseases	Sal-miR-58	Modulates PFKP expression or activity	–	Yang et al., 2023
Compounds studied only in non-neoplastic diseases	Indoxyl sulfate	Modulates PFKP expression or activity	Renal fibrosis, inflammation	Qin et al., 2020

## 9 Summary and prospect of PFKP in cancer research

### 9.1 Summary

Platelet-type phosphofructokinase (PFKP), an essential isoform of phosphofructokinase-1 (PFK-1), serves as a pivotal metabolic node linking glucose metabolic reprogramming with the progression of malignant tumors. Recent studies have challenged the traditional view of PFKP as merely a rate-limiting enzyme in glycolysis, revealing its diverse roles in metabolic regulation, signal transduction, transcriptional modulation, and remodeling of the tumor microenvironment. Comprehensive pan-cancer analyses and clinical investigations have shown that aberrant expression of PFKP is prevalent across a wide range of malignancies and is closely associated with tumor proliferation, invasion, metastasis, therapeutic resistance, and patient prognosis. These findings suggest the potential of PFKP as a biomarker for tumor diagnosis, prognostic evaluation, and prediction of therapeutic response.

At the molecular mechanistic level, PFKP supports an enhanced glycolytic phenotype, providing energy and biosynthetic precursors essential for the rapid proliferation of tumor cells, and acting as a pivotal effector molecule in promoting the Warburg effect. However, a growing body of recent research indicates that the functions of PFKP extend significantly beyond traditional metabolic roles. Phosphofructokinase platelet-type (PFKP) plays a pivotal role in tumorigenesis by promoting tumor cell proliferation, invasion, maintenance of stemness, and therapeutic resistance through the modulation of signaling pathways such as β-catenin, NF-κB, and PI3K/AKT. Conversely, the expression and functional activity of PFKP are intricately regulated by multiple mechanisms, including transcription factors, epigenetic modifications, ubiquitin-mediated degradation, and various post-translational modifications. Furthermore, the nuclear translocation of PFKP enables it to perform non-canonical functions, such as participating in DNA damage repair, transcriptional regulation, and the adaptive evolution of tumors. Additionally, PFKP is increasingly recognized as a critical mediator linking intracellular metabolic reprogramming with the modulation of the tumor microenvironment. It facilitates immune evasion and therapeutic resistance by regulating immune cell recruitment, sustaining cancer stem cell populations, and mediating intercellular metabolic communication.

In the context of clinical translation, PFKP, recognized as a key regulator in tumor metabolism, has been identified as a promising target for metabolism-directed therapeutic strategies. To date, various intervention strategies targeting PFKP, such as small-molecule inhibitors, natural bioactive compounds, and pharmacological approaches that modulate PFKP expression and stability, have exhibited potential anti-tumor effects in preclinical studies. However, due to the intricate and dynamically adaptable nature of tumor metabolic networks, therapies targeting PFKP remain in the exploratory stages, and their clinical translational potential requires further rigorous validation.

### 9.2 Limitations of current studies

Despite significant advancements in elucidating the roles of PFKP in tumorigenesis and tumor progression, several pivotal scientific questions remain unanswered. Firstly, current research predominantly emphasizes the cell-autonomous metabolic regulation of tumor cells by PFKP, while its roles within the complex tumor microenvironment have not been systematically characterized. The expression patterns and functional status of PFKP in various immune cells, fibroblasts, and vascular-associated cells, along with the mechanisms underlying intercellular metabolic crosstalk, are insufficiently defined. This gap in knowledge impedes a comprehensive understanding of the PFKP-mediated immune regulatory network. Secondly, the functions of PFKP demonstrate significant tumor heterogeneity and subtype specificity. The regulatory patterns and functional outputs of PFKP exhibit significant variation across different cancer types and pathological stages; however, systematic comparative studies and mechanistic analyses remain limited. Moreover, the majority of existing evidence is based on fundamental laboratory experiments and retrospective clinical analyses. There is a notable absence of large-sample, multicenter prospective clinical trials necessary to confirm the efficacy and reliability of PFKP as a biomarker for tumor diagnosis and prognosis.

In the realm of PFKP-targeted therapies, significant challenges continue to impede progress. Firstly, PFKP exhibits substantial structural homology with its isoforms, PFKL and PFKM. Consequently, pan-PFK inhibitors demonstrate limited selectivity and are associated with significant off-target effects, while the advancement of isoform-specific therapeutic agents remains considerably underdeveloped. Secondly, tumor metabolism is marked by compensatory reprogramming; as a result, targeting PFKP with a single agent often triggers compensatory activation of alternative metabolic pathways, thereby diminishing therapeutic efficacy. Furthermore, the regulatory mechanisms involving post-translational modifications and dynamic subcellular localization that govern PFKP functions have yet to be fully elucidated. Additionally, the comprehensive mapping of its upstream and downstream regulatory networks is incomplete, significantly hindering its translation into clinical applications.

It should be noted that this is a traditional narrative review instead of a formal systematic review. Several inherent methodological limitations should be acknowledged. First, without a pre-registered protocol, our literature retrieval strategy cannot be fully reproduced. Second, potential publication bias may exist because studies reporting positive findings tend to be published and cited more frequently.

### 9.3 Research prospects

Future research on PFKP within the field of oncology is poised to focus on three primary directions: comprehensive mechanistic investigation, technological advancements, and clinical translation. Regarding fundamental mechanistic research, a more detailed examination of the non-canonical functions of PFKP is essential, with an emphasis on elucidating its regulatory mechanisms related to nuclear localization, patterns of epigenetic modification, and the complex regulatory networks through which it influences tumor metabolic reprogramming, maintenance of cancer stem cell stemness, and immune evasion. Concurrently, a systematic analysis of the functional heterogeneity of PFKP across various cancer types and under different therapeutic stresses is necessary to elucidate its diverse roles at various stages of tumor progression and to establish a comprehensive molecular framework underlying PFKP-driven malignant progression. Furthermore, an in-depth investigation into the interaction mechanisms between PFKP and various cellular components within the tumor microenvironment may reveal novel targets for overcoming tumor drug resistance and remodeling immunosuppressive microenvironments.

In the context of targeted therapeutic development, researchers can exploit the isoform-specific structural characteristics of PFKP to design highly selective and low-toxicity small-molecule inhibitors. This approach aims to mitigate the off-target effects associated with existing pan-metabolic inhibitors. Given the compensatory mechanisms inherent in tumor metabolism, it is imperative to investigate combinatorial regimens that pair PFKP-targeted agents with inhibitors of glycolysis, glutamine metabolism, and other metabolic pathways. Such strategies could effectively obstruct compensatory metabolic reprogramming in tumors and enhance anti-tumor efficacy. Furthermore, the exploration of synergistic combination regimens that integrate PFKP-targeted strategies with chemotherapy, molecular targeted therapy, and immunotherapy is essential. This approach holds promise for overcoming multi-drug resistance in tumors and enriching the comprehensive cancer treatment arsenal.

For the clinical translational application of PFKP, it is imperative to conduct large-scale, multicenter prospective clinical trials to validate its clinical utility as a pan-cancer non-invasive biomarker for diagnosis, prognostic stratification, and therapeutic response prediction. These trials should also aim to develop an individualized tumor diagnosis and treatment evaluation system based on PFKP expression levels. By integrating multi-omics technologies, PFKP-related molecular signature profiles can be identified to assess its potential application in early tumor screening and recurrence monitoring. With ongoing in-depth mechanistic studies and advancements in targeted technologies, PFKP holds promise as a novel biomarker for precise tumor diagnosis and as an effective and safe therapeutic target. This could provide innovative strategies for clinical precision oncology, reversal of drug resistance, and improved patient prognosis.

## References

[B1] Bolaños-Suárez V. Alfaro A. Espinosa A. M. Medina-Martínez I. Juárez E. Villegas-Sepúlveda N. (2022). Gene expression levels of the glycolytic enzymes lactate dehydrogenase A (LDHA) and phosphofructokinase platelet (PFKP) are good predictors of survival time, recurrence and risk of death in cervical. Cancer. 10.1101/2022.08.02.22277946 PMC10417302 37326348

[B2] Bolanos-Suarez V. Alfaro A. Espinosa A. M. Medina-Martinez I. Juarez E. Villegas-Sepulveda N. (2023). The mRNA and protein levels of the glycolytic enzymes lactate dehydrogenase A (LDHA) and phosphofructokinase platelet (PFKP) are good predictors of survival time, recurrence, and risk of death in cervical cancer patients. Cancer Med. 12 (14), 15632–15649. 10.1002/cam4.6123 PMC10417302 37326348

[B3] Broxmeyer H. E. (2021). All in for nuclear PFKP-induced CXCR4 metastasis: a T cell acute lymphoblastic leukemia prognostic marker. J. Clin. Invest. 131, e151295. 10.1172/jci151295 PMC8363282 34396983

[B4] Calvier L. Chouvarine P. Legchenko E. Hoffmann N. Geldner J. Borchert P. (2017). PPARγ links BMP2 and TGFβ1 pathways in vascular smooth muscle cells, regulating cell proliferation and glucose metabolism. Cell. Metab. 25 5 (5), 1118–1134. 10.1016/j.cmet.2017.03.011 28467929

[B5] Campos M. Albrecht L. V. (2023). Hitting the sweet spot: how glucose metabolism is orchestrated in space and time by Phosphofructokinase-1. Cancers. (Basel) 16 (1), 16. 10.3390/cancers16010016 PMC10778546 38201444

[B6] Cao L. Wang R. Liu G. Zhang Y. Thorne R. F. Zhang X. D. (2023). Glycolytic Pfkp acts as a Lin41 protein kinase to promote endodermal differentiation of embryonic stem cells. EMBO Rep. 24 (3), e55683. 10.15252/embr.202255683 PMC9986826 36660859

[B7] Cao C. Lv C. ElFar A. (2026). Targeting phosphofructokinase in cancer: integrating natural products for metabolic reprogramming and therapeutic innovation. Front. Pharmacol. 17, 1844992. 10.3389/fphar.2026.1844992 PMC13291013 42358350

[B8] Cengiz B. (2024). The importance of PFK-P and PEA-15 (Phosphoprotein enriched in astrocytes-15kDa) expression in cases of endometrioid endometrial carcinoma and endometrial atypical hyperplasia/endometrioid intraepithelial neoplasia. Virchows. Arch. 485, S278–S279.

[B9] Cengiz B. B. Arik D. Oge T. Yalcin O. T. Ozen A. (2024). The Importance of PFK-P (Phosphofructokinase-Platelet) and PEA-15 (Phosphoprotein Enriched in Astrocytes-15Kda) Expression in Cases of Endometrioid Endometrial Carcinoma and Endometrial Atypical hyperplasia/endometrioid Intraepithelial Neoplasia, 485.

[B10] Chen g. Zhang X. (2023). Glycolysis regulator PFKP induces human melanoma cell proliferation and tumor growth. Clin. Transl. Oncol. 25 (7), 2183–2191. 10.1007/s12094-023-03096-7 36792847

[B11] Chen C. Liao D. Wang J. Liang Z. Yao Q. (2014). Anti-human protein S antibody induces tissue factor expression through a direct interaction with platelet phosphofructokinase. Thromb. Res. 133 (2), 8–222. 10.1016/j.thromres.2013.11.009 24331211 PMC3947162

[B12] Chen G. Liu H. Zhang Y. Liang J. Zhu Y. Zhang M. (2018). Silencing PFKP inhibits starvation-induced autophagy, glycolysis, and epithelial mesenchymal transition in oral squamous cell carcinoma. Exp. Cell. Res. 370 (1), 46–57. 10.1016/j.yexcr.2018.06.007 29894707

[B13] Chen L. Hu N. Wang C. Zhao H. (2020). HOTAIRM1 knockdown enhances Cytarabine-Induced cytotoxicity by suppression of glycolysis through the Wnt/β-catenin/PFKP pathway in acute myeloid. Leuk. Cells, 680. 10.1016/j.abb.2019.108244 31904363

[B14] Chen J. Zou L. Lu G. Grinchuk O. Fang L. Ong D. S. T. (2022). PFKP alleviates glucose starvation-induced metabolic stress in lung cancer cells *via* AMPK-ACC2 dependent fatty acid oxidation. Cell. Discov. 8, 52. 10.1038/s41421-022-00406-1 PMC9156709 35641476

[B15] Chen F. Tang C. Yang F. Ekpenyong A. Qin R. Xie J. (2024). HSP90 inhibition suppresses tumor glycolytic flux to potentiate the therapeutic efficacy of radiotherapy for head and neck cancer. Sci. Adv. 10 (8), eadk3663. 10.1126/sciadv.adk3663 PMC10889358 38394204

[B16] Chen X. Wang Y. Wan J. Dou X. Zhang C. Sun M. (2024a). Quercetin alleviates liver fibrosis *via* regulating glycolysis of liver sinusoidal endothelial cells and neutrophil infiltration. Biomol. and Biomedicine 24 (6), 1806–1815. 10.17305/bb.2024.10530 PMC11496877 38943679

[B17] Chen X. Su W. Chen J. Ouyang P. Gong J. (2024b). RNF123 inhibits cell viability, cell cycle and colony formation of breast cancer by inhibiting glycolysis *via* ubiquitination of PFKP. Naunyn-Schmiedeberg's Archives Pharmacol. 398, 7219–7233. 10.1007/s00210-024-03723-2 39725718

[B18] Cheng L. Qin T. Ma J. Duan W. Xu Q. Li X. (2019). Hypoxia-inducible Factor-1α mediates hyperglycemia-induced pancreatic cancer glycolysis. Anticancer. Agents Med. Chem. 19, 1503–1512. 10.2174/1871520619666190626120359 31241439

[B19] Cheng Z. Huang H. Li M. Chen Y. (2024). Proteomic analysis identifies PFKP lactylation in SW480 colon cancer cells. iScience 27 (1), 108645. 10.1016/j.isci.2023.108645 PMC10753066 38155775

[B20] Cho E. S. Kim N. H. Yun J. S. Cho S. B. Kim H. S. Yook J. I. (2020). Breast cancer subtypes underlying EMT-Mediated catabolic metabolism. Cells 9 (9), 2064. 10.3390/cells9092064 32927665 PMC7563728

[B21] Dai S. Venturini E. Yadav S. Lin X. Clapp D. Steckiewicz M. (2022). Calcium/calmodulin-dependent protein kinase kinase 2 mediates pleiotropic effects of epidermal growth factor in cancer cells. Biochimica biophysica acta. Mol. Cell. Res. 1869 (7), 119252. 10.1016/j.bbamcr.2022.119252 35271909

[B22] Deng L. Zhao Y. Liu W. (2023). PFKP is upregulated in 5-fluorouracil-resistant patients and suppresses the antitumor activity of 5-fluorouracil in colorectal cancer *in vitro* and *in vivo* . J. Chemother. 36, 1–13. 10.1080/1120009x.2023.2288742 38044588

[B23] Eto K. Sakura H. Yasuda K. Hayakawa T. Kawasaki E. Moriuchi R. (1994). Cloning of a complete protein-coding sequence of human platelet-type phosphofructokinase isozyme from pancreatic islet. Biochem. Biophys. Res. Commun. 198 (3), 990–998. 10.1006/bbrc.1994.1141 8117307

[B24] Fan Y. Wang J. Xu Y. Wang Y. Song T. Liang X. (2021). Anti-Warburg effect by targeting HRD1-PFKP pathway May inhibit breast cancer progression. Cell. Communication Signaling CCS 19 (1), 18. 10.1186/s12964-020-00679-7 PMC7883444 33588886

[B25] Fang K. Ma Y. Li h. Yue Y. Ruan H. Xiong S. (2026). PFKP is required for chemoresistant phenotype of breast cancer through modulating the formation of CD133+ cancer stem like cells. Mol. Biomed. 7 (1), 56. 10.1186/s43556-026-00454-z PMC13106758 42024199

[B26] Fendt S. Frezza C. Erez A. (2020). Targeting metabolic plasticity and flexibility dynamics for cancer therapy. Cancer Discov. 10 (12), 1797–1807. 10.1158/2159-8290.cd-20-0844 33139243 PMC7710573

[B27] Fernandes P. M. Kinkead J. McNae I. Michels P. A. Walkinshaw M. D. (2020). Biochemical and transcript level differences between the three human phosphofructokinases show optimisation of each isoform for specific metabolic niches. Biochem. J. 477 (22), 4425–4441. 10.1042/bcj20200656 33141153 PMC7702303

[B28] Fujiwara H. Tateishi K. Misumi K. Hayashi A. Igarashi K. Kato H. (2019). Mutant IDH1 confers resistance to energy stress in normal biliary cells through PFKP-induced aerobic glycolysis and AMPK activation. Sci. Rep. 9 (1), 18859. 10.1038/s41598-019-55211-w 31827136 PMC6906335

[B29] Fukushi A. Kim H. Chang Y. Kim C. (2022). Revisited metabolic control and reprogramming cancers by means of the warburg effect in tumor cells. Int. J. Mol. Sci. 23, 10037. 10.3390/ijms231710037 PMC9456516 36077431

[B30] Ganapathy-Kanniappan S. (2020). PFKP phenotype in lung cancer: prognostic potential and beyond. Mol. Biol. Rep. 47 (10), 8271–8272. 10.1007/s11033-020-05805-9 32915402

[B31] Gandhirajan A. Roychowdhury S. Kibler C. Cross E. Abraham S. Bellar A. (2023). SIRT2-PFKP interaction dysregulates phagocytosis in macrophages with acute ethanol-exposure. Front. Immunol. 13, 1079962. 10.3389/fimmu.2022.1079962 PMC9972587 36865524

[B32] Gao X. Qin S. Wu Y. Chu C. Jiang B. Johnson R. H. (2021). Nuclear PFKP promotes CXCR4-dependent infiltration by T cell acute lymphoblastic leukemia. J. Clin. Invest. 131 (16), e143119. 10.1172/jci143119 PMC8363288 34255748

[B33] Ge H. Du Z. Liu W. Wang L. Li J. Yang G. (2025). Gen-miR-5 derived from Gentianella acuta inhibits PFKP to prevent fibroblast activation and alleviate myocardial fibrosis. Front. Pharmacol. 16, 1578877. 10.3389/fphar.2025.1578877 PMC12081263 40385478

[B34] Gong C. Yang M. Long H. Liu X. Xu Q. Qiao L. (2024). IL-6-Driven autocrine lactate promotes immune escape of uveal melanoma. Investigative Opthalmology and Vis. Sci. 65 (3), 37. 10.1167/iovs.65.3.37 PMC10981435 38551584

[B35] Guddeti R. K. Pacharla H. Yellapu N. K. Karyala P. Pakala S. B. (2023). MORC2 and MAX contributes to the expression of glycolytic enzymes, breast cancer cell proliferation and migration. Med. Oncology N. Lond. Engl. 40 (3), 102. 10.1007/s12032-023-01974-2 36802305

[B36] Guo D. Meng Y. Zhao G. Wu Q. Lu Z. (2025). Moonlighting functions of glucose metabolic enzymes and metabolites in cancer. Nat. Rev. Cancer. 25 (6), 426–446. 10.1038/s41568-025-00800-3 40175621

[B37] Halford S. E. R. Walter H. McKay P. Townsend W. Linton K. Heinzmann K. (2021). Phase I expansion study of the first-in-class monocarboxylate transporter 1 (MCT1) inhibitor AZD3965 in patients with diffuse large B-cell lymphoma (DLBCL) and Burkitt lymphoma (BL). J. Clin. Oncol. 39 (15_Suppl. l), 3115. 10.1200/jco.2021.39.15_suppl.3115

[B38] Halford S. Veal G. J. Wedge S. R. Payne G. S. Bacon C. M. Sloan P. (2023). A phase I dose-escalation study of AZD3965, an oral monocarboxylate transporter 1 inhibitor, in patients with advanced cancer. Clin. Cancer. Res. 29 (8), 1429–1439. 10.1158/1078-0432.ccr-22-2263 PMC7614436 36652553

[B39] Han L. He J. Xie H. Gong Y. Xie C. (2024). Pan-cell death-related signature reveals tumor immune microenvironment and optimizes personalized therapy alternations in lung adenocarcinoma. Sci. Rep. 14 (1), 15682. 10.1038/s41598-024-66662-1 PMC11231366 38977778

[B40] Holohan C. Schaeybroeck S. V. Longley D. B. Johnston P. G. (2013). Cancer drug resistance: an evolving paradigm. Nat. Rev. Cancer. 13 (10), 714–726. 10.1038/nrc3599 24060863

[B41] Huang Y. Xiong C. Wang C. Deng J. Zuo Z. Wu H. (2023). P53-responsive CMBL reprograms glucose metabolism and suppresses cancer development by destabilizing phosphofructokinase PFKP. Cell. Rep. 42 (11), 113426. 10.1016/j.celrep.2023.113426 37967006

[B42] Inaishi T. Shibata M. Ichikawa T. Kanda M. Hayashi M. Soeda I. (2022). Platelet isoform of phosphofructokinase accelerates malignant features in breast cancer. Oncol. Rep. 47 (1), 9. 10.3892/or.2021.8220 34751415

[B43] Ishaq A. R. Younis T. Lin S. Usman M. Wang T. Chen Z. (2024). Phosphofructokinase-1 in cancer: a promising target for diagnosis and therapy. Recent. Pat. anticancer. Drug. Discov. 20, 667–680. 10.2174/0115748928321372240813080131 39171598

[B44] Jeon S. M. Lim J. S. Park S. H. Lee J. (2021). Wnt signaling promotes tumor development in part through phosphofructokinase 1 platelet isoform upregulation. Oncol. Rep. 46 (5), 234. 10.3892/or.2021.8185 34515327

[B45] Jeong S. Park S. Choi J. Cho N. Moon J. Gil H. (2024). Indoxyl sulfate induces apoptotic cell death by inhibiting glycolysis in human astrocytes. Kidney Res. Clin. Pract. 43, 774–784. 10.23876/j.krcp.23.005 PMC11615446 37956994

[B46] Jiang Z. Liu T. Wang Y. Li J. Guo L. (2024). Effect of lncRNA XIST on acute myeloid leukemia cells *via* miR-142-5p-PFKP axis. Hematol. Amst. Neth. 29 (1), 2306444. 10.1080/16078454.2024.2306444 38305210

[B47] JONG-HOLEE (2023). Phosphofructokinase 1 platelet isoform enhances VEGF expression in part through HIF-la Up-regulation in breast cancer. Anticancer Res. Int. J. Cancer Res. Treat. 10 (1), 75–84. 10.21873/anticanres.16136 36585185

[B48] Kahn A. Meienhofer M. Cottreau D. Lagrange J. Dreyfus J. (1979). Phosphofructokinase (PFK) isozymes in man: I. Studies of adult human tissues. Hum. Genet. 48 (1), 93–108. 10.1007/bf00273280 156693

[B49] Kanai S. Shimada T. Narita T. Okabayashi K. (2019). Phosphofructokinase-1 subunit composition and activity in the skeletal muscle. Liver, Brain Dogs 81, 712–716. 10.1292/jvms.19-0049 30918224 PMC6541852

[B50] Kloos M. Bruser A. Kirchberger J. Schoneberg T. Strater N. (2015). Crystal structure of human platelet phosphofructokinase-1 locked in an activated conformation. Biochem. Journal 469 (3), 32–421. 10.1042/BJ20150251 26205495

[B51] Korotaeva A. Borunova A. Kuzevanova A. Zabotina T. Alimov A. (2023). Molecular mechanisms of impaired antigenic presentation as a cause of tumor escape from immune surveillance. Arkhiv Patologii 85 (6), 76. 10.17116/patol20238506176 38010642

[B52] Lang L. Chemmalakuzhy R. Shay C. Teng Y. (2019). PFKP signaling at a glance: an emerging mediator of cancer cell metabolism. Adv. Exp. Med. Biol. 1134, 243–258. 10.1007/978-3-030-12668-1_13 30919341

[B53] Lee J. Liu R. Li J. Zhang C. Wang Y. Cai Q. (2017). Stabilization of phosphofructokinase 1 platelet isoform by AKT promotes tumorigenesis. Nat. Commun. 8 (1), 949. 10.1038/s41467-017-00906-9 29038421 PMC5643558

[B54] Lee J. Liu R. Li J. Wang Y. Tan L. Li X. (2018). EGFR-phosphorylated platelet isoform of phosphofructokinase 1 promotes PI3K activation. Mol. Cell. 70 (2), 197–210.e7. 10.1016/j.molcel.2018.03.018 29677490 PMC6114939

[B55] Lee J. Shao F. Ling J. Lu S. Liu R. Du L. (2020). Phosphofructokinase 1 platelet isoform promotes beta-Catenin transactivation for tumor development. Front. Oncol. 10, 211. 10.3389/fonc.2020.00211 32195176 PMC7066116

[B56] Lei Z. Tian Q. Teng Q. Wurpel J. N. D. Zeng L. Pan Y. (2023). Understanding and targeting resistance mechanisms in cancer. MedComm 4 (3), e265. 10.1002/mco2.265 PMC10203373 37229486

[B57] Li X. Sun L. Yan G. Yan X. (2021). PFKP facilitates ATG4B phosphorylation during amino acid deprivation-induced autophagy. Cell. Signal. 82, 109956. 10.1016/j.cellsig.2021.109956 33607258

[B58] Li Z. Yue M. Liu X. Liu Y. Lv L. Zhang P. (2022). The PCK2-glycolysis axis assists three-dimensional-stiffness maintaining. Stem Cell. Osteogenes. 18, 492–506. 10.1016/j.bioactmat.2022.03.036 PMC8971594 35415308

[B59] Li, C. Yeh Y. Tsai Y. Huang T. Shen M. Lin P. (2023). Controlling the confounding effect of metabolic gene expression to identify actual metabolite targets. Microsatellite Instab. Cancers 17, 18. 10.1186/s40246-023-00465-9 PMC9990231 36879264

[B60] Li Q. Miao J. Shi N. Lin C. Hu X. Shen Y. (2023). The lncRNA XIST/miR-29b-3p/COL3A1 axis regulates central carbon metabolism in head and neck squamous cell carcinoma and is associated with poor tumor prognosis. Ann. Transl. Med. 11 (4), 165. 10.21037/atm-23-30 PMC10009572 36923098

[B61] Li S. Fang Y. Jiang L. Zhang C. Wang Y. Chen S. (2025). TRIM25 potentiates innate immune response to senecavirus A by enhancing K63-linked ubiquitination of RIG-I and K48-linked ubiquitin-facilitated degradation of PFKP. Vet. Microbiol. 308, 110634. 10.1016/j.vetmic.2025.110634 40651151

[B62] Li Z. Chen P. Lin Y. Zhang J. Ding J. Ahmed R. Z. (2025). Inhibition of Platelet Activation Process upon Tris (2-chloroethyl) Phosphate Exposure: Role of PFKP-mediated Glycolysis and the Pentose Phosphate Pathway. 10.1016/j.envpol.2025.125714 39828204

[B63] Liao D. Wang J. Lin P. Yao Q. Chen C. (2009). QS418. anti-human protein S antibody induces tissue factor expression through direct interaction with platelet phosphofructokinase in human coronary artery endothelial cells. J. Surg. Res. 151 (2), 301. 10.1016/j.jss.2008.11.730

[B64] Lim J. S. Shi Y. Park S. H. Jeon S. M. Zhang C. Park Y. (2022). Mutual regulation between phosphofructokinase 1 platelet isoform and VEGF promotes glioblastoma tumor growth. Cell. death. Dis. 13 (11), 1002. 10.1038/s41419-022-05449-6 PMC9701207 36435833

[B65] Lincet H. Icard P. (2014). How do glycolytic enzymes favour cancer cell proliferation by nonmetabolic functions? Oncogene 34, 3751–3759. 10.1038/onc.2014.320 25263450

[B66] Ling X. Zhang L. Fang C. Liang H. Ma J. (2024). A comprehensive prognostic and immunological implications of PFKP in pan-cancer. Cancer Cell. Int. 24 (1), 310. 10.1186/s12935-024-03497-w PMC11385129 39252014

[B67] Liu L. Wang Y. Bai R. Yang K. Tian Z. (2016). MiR-186 inhibited aerobic glycolysis in gastric cancer *via* HIF-1α regulation. Oncogenesis 5, e224. 10.1038/oncsis.2016.35 27159677 PMC4945752

[B68] Liu B. Li F. Liu M. Xu Z. Gao B. Wang Y. (2021). Prognostic roles of phosphofructokinase platelet in clear cell renal cell carcinoma and correlation with immune infiltration. Int. J. Gen. Med. 14, 3645–3658. 10.2147/IJGM.S321337 PMC8312753 34321910

[B69] Liu J. Peng X. Yang S. Li X. Huang M. Wei S. (2022). Extracellular vesicle PD-L1 in reshaping tumor immune microenvironment: biological function and potential therapy strategies. Cell. Commun. Signal. 20 (1), 14. 10.1186/s12964-021-00816-w PMC8796536 35090497

[B70] Liu W. Ding Z. Tao Y. Liu S. Jiang M. Yi F. (2024). A positive feedback loop between PFKP and c-Myc drives head and neck squamous cell carcinoma progression. Mol. Cancer. 23 (1), 141. 10.1186/s12943-024-02051-6 PMC11232239 38982480

[B71] Liu C. Jiang L. Wang P. Hu Y. Zheng Y. Lu L. (2026a). Lactylation of PFKP-K688 enhances glycolytic flux and confers cardioprotection in myocardial ischemia. Front. Pharmacol. 17, 1717779. 10.3389/fphar.2026.1717779 PMC13033730 41919225

[B72] Liu H. Zhang M. Xu Z. Yang Z. Pan Y. Bai Y. (2026). ATM promotes PFKP nuclear translocation to balance glycolysis and homologous recombination repair. Nucleic. acids. Res. 54 (7), gkag336. 10.1093/nar/gkag336 42011782 PMC13096807

[B73] Liu C. Zhang Z. Feng R. Zeng M. Li H. Zhu M. (2026b). Isoliquiritigenin impedes breast cancer progression through PITX1-PFKP-Mediated glycolysis reprogramming. Oncol. Res. 34 (5), 36–10. 10.32604/or.2026.077059 PMC13126404 42065039

[B74] Lu T. Yang Y. F. Cheng C. F. Tu Y. T. Chen Y. R. Lee M. C. (2023). Overexpression accelerated colorectal cancer cell growth and motility. J. Cancer 14 (6), 943–951. 10.7150/jca.82738 37151384 PMC10158518

[B75] Lu T. Yang Y. Cheng C. Tu Y. Chen Y. Lee M. (2023). Phosphofructokinase platelet overexpression accelerated colorectal cancer cell growth and motility. J. Cancer 14 (6), 943–951. 37151384 10.7150/jca.82738 PMC10158518

[B76] Luo X. Zheng D. Zheng R. Wang C. Xu L. Tan H. (2018). The platelet isoform of phosphofructokinase in acute myeloid leukemia: clinical relevance and prognostic implication. Blood 132 (Suppl. 1), 5251. 10.1182/blood-2018-99-113786

[B77] Lynch E. M. Hansen H. Salay L. Cooper M. Timr S. Kollman J. M. (2024). Structural basis for allosteric regulation of human phosphofructokinase-1. Nat. Commun. 15 (1), 7323. 10.1038/s41467-024-51808-6 39183237 PMC11345425

[B78] Mi J. Zhao L. Shen Y. Mo S. Kuang Y. (2024). PFKP Lactylation Promotes the Ovarian Cancer Progression Through Targeting PTEN. 10.1007/s10528-024-10990-4 39638933

[B79] Mo Z. Yu L. Cao Z. Hu H. Luo S. Zhang S. (2020). Identification of a hypoxia-associated signature for lung adenocarcinoma. Front. Genet. 11, 647. 10.3389/fgene.2020.00647 32655624 PMC7324800

[B80] Mo Z. Liu D. Chen Y. Luo J. Li W. Liu J. (2022). Single-cell transcriptomics reveals the role of macrophage-Naïve CD4 + T cell interaction in the immunosuppressive microenvironment of primary liver carcinoma. J. Transl. Med. 20 (1), 466. 10.1186/s12967-022-03675-2 36221095 PMC9552358

[B81] Monika G. Helen T. Yuen-Li C. M c. P. Manuel M. Xiaoke Y. (2011). Adaptation to HIF-1 deficiency by upregulation of the AMP/ATP ratio and phosphofructokinase activation in hepatomas. BMC Cancer 11 (1), 198. 10.1186/1471-2407-11-198 21612605 PMC3123325

[B82] Moon J. Kim H. E. Koh E. Park S. H. Jin W. Park B. (2011). Krüppel-like factor 4 (KLF4) activates the transcription of the gene for the platelet isoform of phosphofructokinase (PFKP) in breast cancer. J. Biological Chemistry 286 (27), 23808–23816. 10.1074/jbc.M111.236737 21586797 PMC3129162

[B83] Moreno-Sánchez R. Marín-Hernández A. Saavedra E. Pardo J. P. Ralph S. J. Rodríguez-Enríquez S. (2014). Who controls the Atp supply in cancer cells? Biochem. Lessons Understand Cancer Energy Metabolism 50, 10–23. 10.1016/j.biocel.2014.01.025 24513530

[B84] Muntz J. A. (1953). Partial purification and some properties of brain phosphofructokinase. Arch. Biochem. Biophys. 42 (2), 435–445. 10.1016/0003-9861(53)90371-3 13031628

[B85] NormaMorrison C. S. Simpson C. Fothergill-Gilmore L. Boyd E. Connor J. M. , (1992). Regional chromosomal assignment of the human platelet phosphofructokinase gene to 10p15. Hum. Genet. 89(1), 105–106. 10.1007/BF00207053 1533608

[B86] Pan C. Li B. Simon M. C. (2021). Moonlighting functions of metabolic enzymes and metabolites in cancer. Mol. Cell. 81 (18), 3760–3774. 10.1016/j.molcel.2021.08.031 34547237

[B87] Pant B. Ahuja A. Roychowdhury S. Shrestha D. Cross E. Wang Y. (2025). Mitoquinol improves phagocytosis and glycolysis in ethanol-exposed macrophages *via* HIF-1α-PFKP axis. J. Immunol. 214, 1754–1772. 10.1093/jimmun/vkaf078 40356076 PMC12311378

[B88] Park Y. Kim S. Han H. Sohn B. Kim J. Liang J. (2013). Tat-activating regulatory DNA-binding protein regulates glycolysis in hepatocellular carcinoma by regulating the platelet isoform of phosphofructokinase through microRNA 520. Hepatology Official J. Am. Assoc. Study Liver Dis. 58 (1), 182–191. 10.1002/hep.26310 23389994 PMC3923572

[B89] Peng J. Li P. Li Y. Quan J. Yao Y. Duan J. (2023). PFKP is a prospective prognostic, diagnostic, immunological and drug sensitivity predictor across pan-cancer. Sci. Rep. 13 (1), 17399. 10.1038/s41598-023-43982-2 37833332 PMC10576092

[B90] Peng Z. Han X. Wang T. Li J. Yang C. Tou F. (2024). PFKP deubiquitination and stabilization by USP5 activate aerobic glycolysis to promote triple-negative breast cancer progression. Breast. Cancer. Res. 26 (1), 10. 10.1186/s13058-024-01767-z 38217030 PMC10787506

[B91] Pires T. R. C. Albanese J. M. Schwab M. Marette A. Carvalho R. S. Sola-Penna M. (2017). Phosphofructokinase-P modulates P44/42 MAPK levels in HeLa cells. J. Cellular Biochemistry 118 (5), 1216–1226. 10.1002/jcb.25774 27791266

[B92] Qin Y. Zheng B. Yang G. Yang H. Zhou J. Yang Z. (2020). Salvia Miltiorrhiza-Derived Sal-miR-58 induces autophagy and attenuates inflammation in vascular smooth muscle. Cells 21, 492–511. 10.1016/j.omtn.2020.06.015 32679544 PMC7360890

[B93] Qing Y. Dong L. Gao L. Li C. Li Y. Han L. (2021). R-2-hydroxyglutarate attenuates aerobic glycolysis in leukemia by targeting the FTO/m(6)A/PFKP/LDHB axis. Mol. Cell. 81 (5), 922–939.e9. 10.1016/j.molcel.2020.12.026 PMC7935770 33434505

[B94] Qiu T. Zhou Y. Hu L. Shan Z. Zhang Y. Fang Y. (2023). 2-Deoxyglucose alleviates migraine-related behaviors by modulating microglial inflammatory factors in experimental model of migraine. Front. Neurol. 14, 1115318. 10.3389/fneur.2023.1115318 37090989 PMC10117646

[B95] Sajjad H. Imtiaz S. Noor T. Siddiqui Y. H. Sajjad A. Zia M. (2021). Cancer models in preclinical research: a chronicle review of advancement in effective cancer research. Anim. Model. Exp. Med. 4 (02), 87–103. 10.1002/ame2.12165 PMC8212826 34179717

[B96] Scherlinger M. Pan W. Hisada R. Boulougoura A. Yoshida N. Vukelic M. (2022). Phosphofructokinase P fine-tunes T regulatory cell metabolism, function, and stability in systemic autoimmunity. Sci. Adv. 8 (48), eadc9657. 10.1126/sciadv.adc9657 36449620 PMC9710877

[B97] Sha X. Wang K. Wang F. Zhang C. Yang L. Zhu X. (2021). Silencing PFKP restrains the stemness of hepatocellular carcinoma cells. Exp. Cell. Res. 407 (1), 112789. 10.1016/j.yexcr.2021.112789 34418458

[B98] Shao S. Qin T. Qian W. Yue Y. Xiao Y. Li X. (2020). Positive feedback in Cav‐1‐ROS signalling in PSCs mediates metabolic coupling between PSCs and tumour. Cells 24, 9397–9408. 10.1111/jcmm.15596 32633891 PMC7417714

[B99] Shen J. Jin Z. Lv H. Jin K. Jonas K. Zhu C. (2020). PFKP is highly expressed in lung cancer and regulates glucose metabolism. Cell. Oncol. 43 (4), 617–629. 10.1007/s13402-020-00508-6 32219704 PMC12990724

[B100] Shi Y. Zhang Z. Di X. Shen G. Tian M. Zhu C. (2026). Integrative analysis of single-cell and bulk transcriptomes reveals metabolic heterogeneity and identifies PFKP as a therapeutic target in cervical cancer. Biochimica biophysica acta. Mol. basis Dis. 1872 (3), 168146. 10.1016/j.bbadis.2025.168146 41448552

[B101] Simpson C. J. Fothergill-Gilmore L. A. (1991). Isolation and sequence of a cDNA encoding human platelet phosphofructokinase. Biochem. Biophys. Res. Commun. 180 (1), 197–203. 10.1016/s0006-291x(05)81276-8 1834056

[B102] Su W. Zhu S. Chen K. Yang H. Tian M. Fu Q. (2021). Overexpressed WDR3 induces the activation of hippo pathway by interacting with GATA4 in pancreatic cancer. J. Exp. Clin. Cancer. Res. 40 (1), 88. 10.1186/s13046-021-01879-w 33648545 PMC7923337

[B103] Su L. Mo R. Dai Y. Wang Z. Lai S. Mo L. (2026). Xueshuantong injection alleviates ischemic heart failure *via* targeting PFKP-mediated endothelial-to-mesenchymal transition. Phytomedicine 155, 158085. 10.1016/j.phymed.2026.158085 41930815

[B104] Sukonina V. Ma H. Zhang W. Bartesaghi S. Subhash S. Heglind M. (2019). FOXK1 and FOXK2 regulate aerobic glycolysis. Nature 566 (Feb.14 TN.7743), 279–283. 10.1038/s41586-019-0900-5 30700909

[B105] Sun C. Xiong D. Yan g. Geng J. Liu M. Yao X. (2016). Genetic alteration in phosphofructokinase family promotes growth of muscle-invasive bladder. Cancer 31, e286–e293. 10.5301/jbm.5000189 26980488

[B106] Sun Y. Zhao H. Feng H. Liu Y. Li Y. Chen S. (2024). PFKP Regulates AXL-MET Oncogenic and Metabolic Pathways in Lung Cancer. 10.1101/2024.03.03.583230

[B107] Sun J. Zhu Q. Shan L. Liu J. (2025). P4HA2 promotes the progression of thyroid cancer by regulating PFKP-mediated glycolysis. J. Physiol. Biochem. 81 (4), 1211–1223. 10.1007/s13105-025-01114-6 40762768

[B108] Tsai M. Y. Kemp R. G. (1973). Isozymes of rabbit phosphofructokinase. J. Biol. Chem. 248 (3), 785–792. 10.1016/s0021-9258(19)44336-6 4265288

[B109] Umar S. M. Kashyap A. Kahol S. Mathur S. R. Gogia A. Deo S. (2020). Prognostic and therapeutic relevance of phosphofructokinase platelet-type (PFKP) in breast cancer. Exp. Cell. Res. 396 (1), 112282. 10.1016/j.yexcr.2020.112282 32919954

[B110] Vigil-Garcia M. Demkes C. J. Eding J. E. C. Versteeg D. de Ruiter H. Perini I. (2021). Gene expression profiling of hypertrophic cardiomyocytes identifies new players in pathological remodelling. Cardiovasc. Res. 117 (6), 1532–1545. 10.1093/cvr/cvaa233 32717063 PMC8152696

[B111] Vora S. Seaman C. Durham S. Piomelli S. (1980). Isozymes of human phosphofructokinase: identification and subunit structural characterization of a new system. Proc. Natl. Acad. Sci. 77 (1), 62–66. 10.1073/pnas.77.1.62 6444721 PMC348208

[B112] Vora S. Halper J. P. Knowles D. M. (1985). Alterations in the activity and isozymic profile of human phosphofructokinase during malignant transformation *in vivo* and *in vitro:* transformation- and progression-linked discriminants of malignancy. Cancer Res. 45 (7), 2993–3001. 3159473

[B113] Voronkova M. A. Hansen H. L. Cooper M. P. Miller J. Sukumar N. Geldenhuys W. J. (2023). Cancer-associated somatic mutations in human phosphofructokinase-1 reveal a critical electrostatic interaction for allosteric regulation of enzyme activity. Biochem. Journal 480 (17), 1411–1427. 10.1042/BCJ20230207 PMC10586780 37622331

[B114] Wang J. Zhang P. Zhong J. Tan M. Ge J. Tao L. (2016). The platelet isoform of phosphofructokinase contributes to metabolic reprogramming and maintains cell proliferation in clear cell renal cell carcinoma. Oncotarget 7 (19), 27142–27157. 10.18632/oncotarget.8382 27049827 PMC5053638

[B115] Wang F. Li L. Zhang Z. (2021). Platelet isoform of phosphofructokinase promotes aerobic glycolysis and the progression of non-small cell lung cancer. Mol. Med. Rep. 23 (1), 74. 10.3892/mmr.2020.11712 33236133 PMC7716410

[B116] Wang D. Liu F. Yang W. Sun Y. Wang X. Sui X. (2022). Meldonium ameliorates hypoxia-induced lung injury and oxidative stress by regulating platelet-type phosphofructokinase-mediated glycolysis. Front. Pharmacol. 13, 863451. 10.3389/fphar.2022.863451 35450040 PMC9017743

[B117] Wang S. Park S. H. Lim J. S. Park Y. Du L. Lee J. (2022). Phosphofructokinase 1 platelet isoform induces PD-L1 expression to promote glioblastoma immune evasion. Genes. Genomics. 44 (12), 1509–1517. 10.1007/s13258-022-01291-4 35917090

[B118] Wang Z. Wu S. Chen X. Liang J. Xue F. Cho W. C. (2023). PFKP confers chemoresistance by upregulating ABCC2 transporter in non-small cell lung cancer. Transl. Lung Cancer Res. 12 (11), 2294–2309. 10.21037/tlcr-23-567 38090515 PMC10713276

[B119] Wang X. Ling R. Peng Y. Qiu W. Chen D. (2024). RNPS1 stabilizes NAT10 protein to facilitate translation in cancer *via* tRNA ac4C modification. 国际口腔科学杂志(英文版) 16 (1), 73–84. 10.1038/s41368-023-00276-7 PMC10800354 38246918

[B120] Wang L. Li H. Yang Y. Shi Q. Feng L. Wang R. (2026). PFKP binding AMOTL1 promotes tumor aerobic glycolysis and epithelial-mesenchymal transition by modulating hippo pathway in head and neck cancer. J. Transl. Int. Med. 14 (1), 108–122. 10.1515/jtim-2026-0006 41727965 PMC12916276

[B121] Wang P. Guo W. Yang Y. Xie Y. Yin F. Li J. (2026). The c-Myc-USP14-PFKP axis sustains chemoresistance in pancreatic ductal adenocarcinoma. Drug Resistance Updates Reviews Commentaries Antimicrobial Anticancer Chemotherapy 86, 101385. 10.1016/j.drup.2026.101385 41812332

[B122] Webb B. A. Forouhar F. Szu F. Seetharaman J. Tong L. Barber D. L. (2015). Structures of human phosphofructokinase-1 and atomic basis of cancer-associated mutations. Nature 523 (7558), 4–111. 10.1038/nature14405 25985179 PMC4510984

[B123] Webb B. A. Dosey A. M. Wittmann T. Kollman J. M. Barber D. L. (2017). The glycolytic enzyme phosphofructokinase-1 assembles into filaments. J. Cell. Biol. 8, 2305–2313. 10.1083/jcb.201701084 28646105 PMC5551713

[B124] Wen Y. Tian M. Jiang X. Gong Y. Gan H. (2024). Trim21 mediates metabolic reprogramming in renal tubular cells *via* PFKP ubiquitination to alleviate renal fibrosis. J. Cell. Physiol. 239 (12), e31439. 10.1002/jcp.31439 39308018

[B125] Wu X. Peng S. Li X. Chen S. Wei Y. Ye Y. (2024). PFKP inhibition protects against pathological cardiac hypertrophy by regulating protein synthesis. Biochimica biophysica acta. Mol. basis Dis. 1871, 167542. 10.1016/j.bbadis.2024.167542 39419453

[B126] Xu W. Zhen Y. Zhou S. Lu N. (2014). Posttranslational regulation of phosphatase and tensin homolog (PTEN) and its functional impact on cancer behaviors. Drug Des. Dev. Ther. 1745, 1745. 10.2147/dddt.s71061 PMC4199979 25336918

[B127] Xu X. Yu Y. Zhang W. Ma W. He C. Qiu G. (2024). SHP-1 inhibition targets leukaemia stem cells to restore immunosurveillance and enhance chemosensitivity by metabolic reprogramming. Nat. Cell. Biol. 26 (3), 464–477. 10.1038/s41556-024-01349-3 38321204

[B128] Xu Z. Wang X. Cheng H. Li J. Zhang X. Wang X. (2025). The role of MCT1 in tumor progression and targeted therapy: a comprehensive review. Front. Immunol. 16, 1610466. 10.3389/fimmu.2025.1610466 40612939 PMC12222217

[B129] Yang S. Wu H. Li Y. Li x. Xiang J. Kang L. (2023). Inhibition of PFKP in renal tubular epithelial cell restrains TGF-β induced glycolysis and renal fibrosis. Cell. death. Dis. 14 (12), 816. 10.1038/s41419-023-06347-1 38086793 PMC10716164

[B130] Yao Y. Yang K. Wang Q. Zhu Z. Li S. Li B. (2024). Prediction of CAF-related genes in immunotherapy and drug sensitivity in hepatocellular carcinoma: a multi-database analysis. Genes. and Immun. 25 (1), 55–65. 10.1038/s41435-024-00252-z PMC10873201 38233508

[B131] Yuan Y. Wang J. Huang L. Guo Y. (2023). Bioinformatics identification of prognostic genes and potential interaction analysis in renal cell carcinoma. Transl. Cancer Res. 12 (4), 774–783. 10.21037/tcr-22-2242 37180655 PMC10174992

[B132] Yuan R. Wang J. Zhang S. Xu Z. Song L. (2025). Phosphofructokinase-1 redefined: a metabolic hub orchestrating cancer hallmarks through multi-dimensional control networks. J. Transl. Med. 23 (1), 873. 10.1186/s12967-025-06897-2 40770805 PMC12330073

[B133] Zancan P. Sola-Penna M. Furtado C. M. Da Silva D. (2010). Differential expression of phosphofructokinase-1 isoforms correlates with the glycolytic efficiency of breast cancer cells. Mol. Genet. Metab. 100 (4), 8–372. 10.1016/j.ymgme.2010.04.006 20483646

[B134] Zhang L. Ke J. Min S. Wu N. Liu F. Qu Z. (2021). Hyperbaric oxygen therapy represses the warburg effect and epithelial–mesenchymal transition in hypoxic NSCLC cells *via* the HIF-1α/PFKP axis. Front. Oncol. 11, 691762. 10.3389/fonc.2021.691762 34367973 PMC8335162

[B135] Zhang Y. Tang Y. Luo Y. Luo L. Shen F. Huang Z. (2021). Triptolide impairs glycolysis by suppressing GATA4/Sp1/PFKP signaling axis in mouse sertoli cells. Toxicol. Appl. Pharmacol. 425, 115606. 10.1016/j.taap.2021.115606 34087332

[B136] Zhang Z. Liang W. Luo Q. Hu H. Yang K. Hu J. (2022). PFKP activation ameliorates foot process fusion in podocytes in diabetic kidney disease. Front. Endocrinol. (Lausanne) 12, 797025. 10.3389/fendo.2021.797025 35095764 PMC8794994

[B137] Zhang L. Jiao G. You Y. Li X. Liu J. Sun Z. (2023). Arginine methylation of PPP1CA by CARM1 regulates glucose metabolism and affects osteogenic differentiation and osteoclastic differentiation. Clin. Transl. Med. 13, e1369. 10.1002/ctm2.1369 37649137 PMC10468565

[B138] Zhang R. Siu M. K. Mo X. Yung M. M. Wang J. Jiang Y. (2023). Abstract 5023: PFKP contributes to ovarian cancer metastasis, stemness and anchorage-independent growth *via* α5 integrin and ERK/MMP9 signaling. Cancer Res. 83 (7_Suppl. ment), 5023. 10.1158/1538-7445.am2023-5023

[B139] Zhang S. Sun H. Hao X. Zhi X. Liu R. Yi T. (2024). Prenatal Glycolysis Restoration can Rescue Myocardial Hypoplasia Caused by Platelet Isoform of Phosphofructokinase 1(PFKP) Deficiency. 10.1101/2024.01.07.24300871

[B140] Zhang W. Xia M. Li J. Liu G. Sun Y. Chen X. (2025). “Warburg effect and lactylation in cancer: mechanisms for chemoresistance,”Mol. Medicine, 31. Cambridge, Mass, 1 146. 10.1186/s10020-025-01205-6 PMC12016192 40264038

[B141] Zhao H. Sun Y. Feng H. Cai J. Liu Y. Li Y. (2024). PFKP silencing suppresses tumor growth *via* the AXL-MET axis. Int. J. Biol. Sci. 20 (15), 6056–6072. 10.7150/ijbs.100525 39664584 PMC11628322

[B142] Zhao H. Shang Z. Sun Y. (2025). Integrating bioinformatics and machine learning to identify glucose metabolism-related biomarkers with diagnostic and prognostic value for patients with kidney renal clear cell carcinoma. Arch. Esp. Urol. 78 (3), 358–370. 10.56434/j.arch.esp.urol.20257803.48 40341002

[B143] Zhong X. He X. Wang Y. Hu Z. Huang H. Zhao S. (2022). Warburg effect in colorectal cancer: the emerging roles in tumor microenvironment and therapeutic implications. J. Hematol. Oncol. 15 (1), 160. 10.1186/s13045-022-01358-5 36319992 PMC9628128

[B144] Zhou K. Yao Y. He Z. Chen C. Zhang X. Yang K. (2018). VDAC2 interacts with PFKP to regulate glucose metabolism and phenotypic reprogramming of glioma stem cells. Cell. death. Dis. 9 (10), 988. 10.1038/s41419-018-1015-x 30250190 PMC6155247

[B145] Zhu J. Chen Y. Jin L. Feng W. Hu a. Xue W. (2023). ZNF148/PFKP/PD-L1 axis promotes non-small cell lung cancer proliferation, migration and glycolysis. J. Cancer Metastasis Treat. 9, 42. 10.20517/2394-4722.2023.111

